# DARPins as a novel tool to detect and degrade p73

**DOI:** 10.1038/s41419-024-07304-2

**Published:** 2024-12-18

**Authors:** Philipp Münick, Jasmin Zielinski, Alexander Strubel, Niklas Gutfreund, Birgit Dreier, Jonas V. Schaefer, Birgit Schäfer, Jakob Gebel, Christian Osterburg, Apirat Chaikuad, Stefan Knapp, Andreas Plückthun, Volker Dötsch

**Affiliations:** 1 Institute of Biophysical Chemistry and Center for Biomolecular Magnetic Resonance, Goethe University, Frankfurt, Germany; 2 Department of Biochemistry, University of Zurich, 8057 Zurich, Switzerland; 3 Institute of Pharmaceutical Chemistry, Goethe University, 60438 Frankfurt, Germany; 4 Structural Genomics Consortium, Goethe University, Frankfurt, Germany

**Keywords:** Biophysical chemistry, Antibody fragment therapy

## Abstract

The concept of Targeted Protein Degradation (TPD) has been introduced as an attractive alternative to the development of classical inhibitors. TPD can extend the range of proteins that can be pharmacologically targeted beyond the classical targets for small molecule inhibitors, as a binding pocket is required but its occupancy does not need to lead to inhibition. The method is based on either small molecules that simultaneously bind to a protein of interest and to a cellular E3 ligase and bring them in close proximity (molecular glue) or a bi-functional molecule synthesized from the chemical linkage of a target protein-specific small molecule and one that binds to an E3 ligase (Proteolysis Targeting Chimeras (PROTAC)). The further extension of this approach to bioPROTACs, in which a small protein-based binding module is fused directly to an E3 ligase or an E3 ligase adaptor protein, makes virtually all proteins amenable to targeted degradation, as this method eliminates the requirement for binding pockets for small molecules. Designed Ankyrin Repeat Proteins (DARPins) represent a very attractive class of small protein-based binding modules that can be used for the development of bioPTOTACS. Here we describe the characterization of two DARPins generated against the oligomerization domain and the SAM domain of the transcription factor p73, a member of the p53 protein family. The DARPins can be used for (isoform-)selective pulldown experiments both in cell culture as well as primary tissue lysates. We also demonstrate that they can be used for staining in cell culture experiments. Fusing them to the speckle type POZ protein (SPOP), an adaptor protein for cullin-3 E3 ligase complexes, yields highly selective and effective degraders. We demonstrate that selective degradation of the ΔNp73α isoform reactivates p53.

## Introduction

Drug development has traditionally focused on small organic molecules. While this approach has been very successful, it also has its limitations. The most severe one is the requirement that the target protein must contain binding pockets for small molecules that also have to be functionally important, thus restricting the effectiveness of this approach to certain protein classes such as enzymes or GPCRs. However, approximately 80% of the human proteome do not contain druggable small molecule binding pockets and are therefore not amenable to inhibition by small organic compounds. The identification of molecular glues [1] and the development of the Proteolysis Targeting Chimeras (PROTAC) approach has in principle made it possible to target also proteins that lack druggable pockets [2–4]. The mechanism of this Targeted Protein Degradation (TPD) is based on small molecules – called molecular glues - binding simultaneously to a target protein and to an E3 ligase, thereby bringing them in close contact. By this mechanism, the target protein gets ubiquitinated and is subsequently degraded by the proteasome. One of the main problems of molecular glue-based approaches is to identify small molecules with a high selectivity. This problem is often circumvented by chemically linking thalidomide derivatives that bind to the E3 ligase Cereblon (or other E3 ligase-specific molecules) with small organic molecules that selectively bind to the target protein. These bi-functional molecules again recruit the target protein to an E3 ligase for initiating degradation. This PROTAC approach, however, still requires the existence of an adequate binding pocket in the target.

An alternative approach is to develop small and highly selective protein-based binders that can target proteins without a binding pocket, such as transcription factors. The Trim-Away approach is based on the selective binding of the Trim21 E3 ligase to the Fc domain of antibodies [5–7], whose natural function is to degrade viruses that have entered the cell with bound antibodies. Microinjection or electroporation of standard antibodies thus leads to the degradation of the target protein that the antibody binds to. An alternative method that can be combined with standard transfection protocols is to fuse nanobodies, monobodies or Designed Ankyrin Repeat Proteins (DARPins) directly to E3 ligases or adaptor proteins of E3 ligases to create so-called bioPROTACs [8–11]. If high affinity and selective natural binding partners exist, they too can be fused to E3 ligases to create selective degraders [12–15]. For example, fusing a peptide (Con1) that binds to the proliferating cell nuclear antigen (PCNA) to the speckle type POZ protein (SPOP), an adaptor protein for cullin-3 (CUL3) was sufficient to efficiently degrade PCNA in HEK293 cells [8].

For the development of target-specific protein-based binders, Designed Ankyrin Repeat Proteins (DARPins) are well suited, as these proteins are small (14-18 kDa), highly stable and, in contrast to antibodies and nanobodies, they do not contain disulfide bonds, making it possible to use them in the reducing cellular environment without reducing their stability [16–18]. DARPins are based on the Ankyrin repeat fold consisting of two antiparallel helices, connected by a loop to the next unit. Three to five repeat units can be combined with specially designed N- and C-terminal capping repeats that shield the hydrophobic core. Positions in the loops connecting the helices as well as on the helix surface can be randomized to create a contiguous concave binding surface. DARPins that bind with high affinity and selectively can be obtained by in vitro selection methods such as ribosome and phage display [19, 20].

Until recently, using biologics as intracellular therapeutics has been hampered by their inability to cross the cellular membrane. Many attempts based on cell penetrating peptide tags and similar concepts [21, 22] have been tested but often without much success. An alternative approach is to deliver the genetic information for the binder or degrader. This can be achieved with virus-like particles using DNA as the genetic material in a cell-specific manner [23]. Since the SARS-CoV-2 pandemic, however, an RNA-based method to achieve cellular expression of proteins has successfully been established as a delivery system: the use of mRNA/lipid nanoparticles [24]. This method works well in certain tissues such as liver, lung or spleen to which nanoparticles can be targeted by specific lipids [25–27] or on the skin and other body surfaces that can be easily accessed. Since for therapeutics, much higher doses and longer durations are needed than for a vaccination, the scope of mRNA/lipid nanoparticles [24] still has to be established. Very recently, it has been demonstrated in the interim analysis of a phase I/II trial that the mRNA/lipid nanoparticle technology can be used to replace a dysfunctional liver enzyme in human patients [28]. This landmark clinical study has also shown that the administration of relatively high doses of mRNA/lipid nanoparticles over long periods of time is in principle safe in humans.

Recently, we have reported the development of DARPins that target all folded domains of the transcription factor p63 [29] as well as the heterotetramer consisting of a p63 dimer and a p73 dimer [30]. Here we report on the development and characterization of DARPins selective for the p73 SAM and the oligomerization domains (OD), thus completing the arsenal of DARPins for the individual domains of p63 and p73, and we demonstrate that these DARPins can be fused to E3 ligases or E3 ligase adaptor proteins to create highly selective and affine bioPROTAC degraders.

## Results

We used a similar selection strategy to find highly selective binders for the OD and the SAM domain of p73 as we had used to develop DARPins for all folded domains of p63 [29] and the hetero-tetramer of p63 and p73 [30] (see Materials and Methods for details). Binders for each domain were selected using ribosome display of the DARPin library, and individual clones were subsequently screened by homogenous time-resolved fluorescence (HTRF) (see Methods). Out of this initial screen, DARPin 1800 was selected as binder for the p73 OD and DARPin B9 as binder for the p73 SAM domain. The previously described non-binding DARPin E3_5 (control DARPin) [31] was used as a negative control for all experiments.

### Affinity and selectivity of binding

**Fig. 1:**
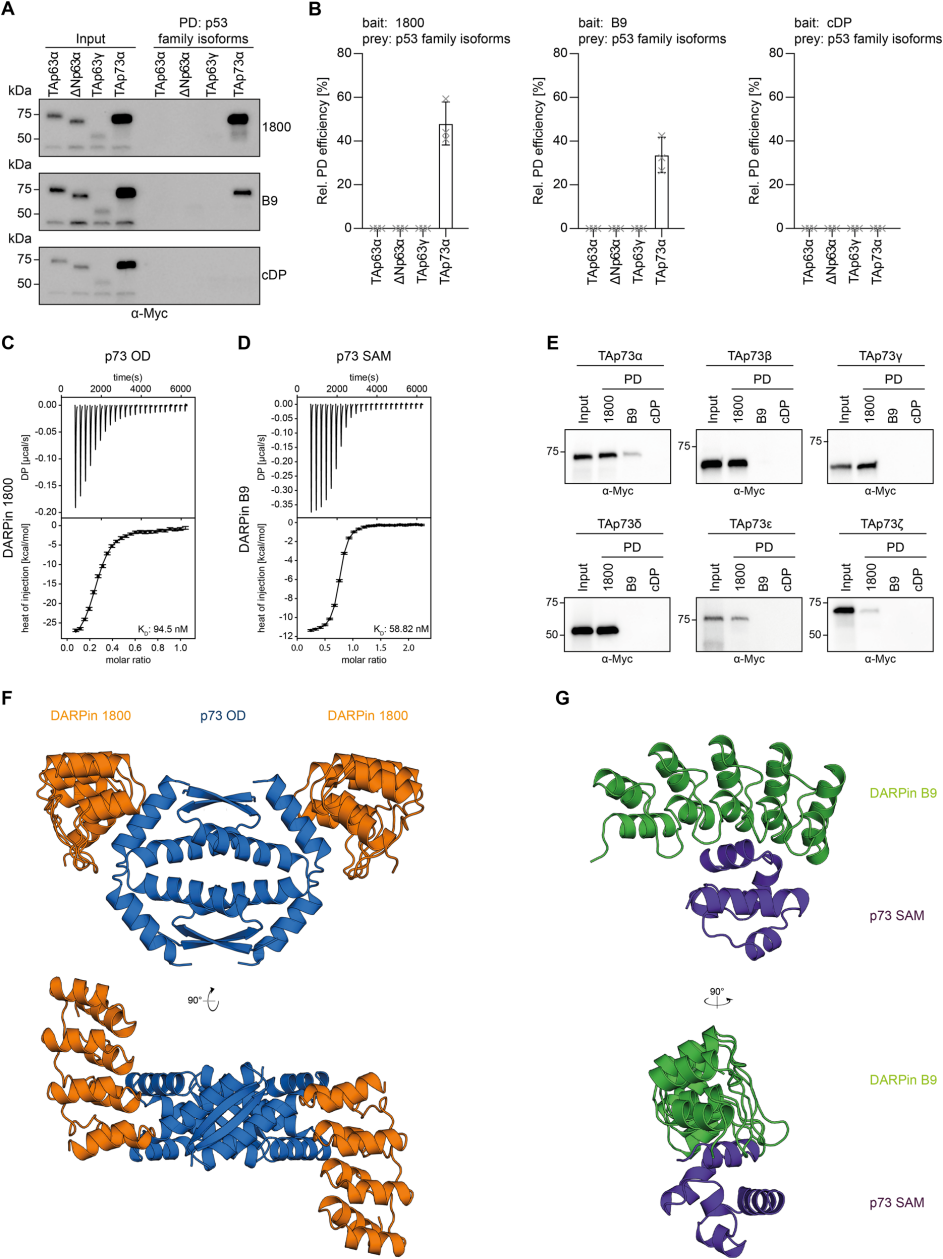
Characterization of DARPins binding to p73 domains. **A** Pulldown experiment with stable HeLa cell lines expressing different Myc-tagged p63 or p73 isoforms. Input signals are shown on the left, signals after pulldown on the right. DARPin 1800 and DARPin B9 bind only to p73 but not to p63 isoforms. **B** Quantification of the pulldown experiments shown in (**A**). The relative pulldown efficiency normalized to the input samples is shown on the *y*-axis. The bar diagram shows the mean values and the error bars show the corresponding SD of three biological replicates. An ordinary one-way ANOVA analysis was performed to assess the statistical significance. **C** Interaction study of the p73 OD and DARPin 1800 using ITC. The top diagram shows the raw measurement and the bottom diagram the integrated heat per titration step. The *K*_D_ value for the interaction is given in the bottom right corner. The measurement was performed at 25 °C. **D** Interaction study of the p73 SAM domain and DARPin B9 using ITC. The top diagram shows the raw measurement and the bottom diagram the integrated heat per titration step. The *K*_D_ value for the interaction is given in the bottom right corner. The measurement was performed at 25 °C. **E** Pulldown experiments with different Myc-tagged p73 isoforms expressed in Rabbit Reticulocyte Lysate (RRL). Input signals are shown on the left, signals after pulldown on the right. The experiments were performed in biological triplicates with exemplary blots of one replicate shown. **F** Crystal structure of DARPin 1800 (orange) in complex with the p73 OD (blue) shown in two different orientations rotated by 90°. **G** Crystal structure of DARPin B9 (green) in complex with the p73 SAM domain (purple) shown in two different orientations rotated by 90°.

To investigate the selectivity of the DARPins we performed pulldown experiments with full-length, Myc-tagged p63 and p73 isoforms overexpressed in HeLa cells using biotinylated DARPins immobilized on Streptavidin beads. The pulldown demonstrated that both lead DARPins selectively bind to p73 but not to p63 (Fig. 1A, B, Supplementary Fig. S1), while no binding was detectable for the control DARPin. Next, we performed isothermal titration calorimetry (ITC) to determine the binding affinity of both DARPins to their respective target domain. For this purpose, the OD of p73 (aa 351–398 of TAp73α) was titrated with DARPin 1800, yielding a dissociation constant of 94.5 nM (Fig. 1C, Supplementary Table S1). Titration of DARPin 1800 with the ODs of p53 and p63 as well as with the p63_2_/p73_2_ heteroOD [32, 33] did not reveal any binding (Supplementary Fig. S2A). Titration of the SAM domain of p73 (aa 489–550 of TAp73α) with DARPin B9 yielded a *K*_D_ of 58.8 nM (Fig. 1D, Supplementary Table S1), while titration with the SAM domain of p63 did not show binding, confirming specificity for p73(Supplementary Fig. S2B). We also titrated the ODs of all p53 family members, the p63_2_/p73_2_ heteroOD and the SAM domains of p63 and p73 with the control DARPin without detecting any binding (Supplementary Fig. S2C, D).

As p73 is expressed in many different isoforms, we wanted to determine the specificity of the DARPins by performing pulldown experiments with Myc-tagged p73 isoforms expressed in Rabbit reticulocyte lysate. While DARPin 1800 detected all C-terminal isoforms, DARPin B9 is a highly specific binder for the α-isoform, being the only one containing the entire SAM domain (Fig. 1E). The control DARPin showed no binding to any isoform. The same pulldown assay was performed with Myc-tagged p73 isoforms transiently overexpressed in H1299 cells, a human non-small cell lung carcinoma cell line. Unfortunately, the input signals of some isoforms (e.g. TAp73γ) are barely visible on the Western Blot, as these transcriptionally active isoforms are rapidly degraded inside cells. Nonetheless, the initial result obtained with Myc-tagged p73 isoforms expressed in RRL has been confirmed (Supplementary Fig. 1E).

### Structural characterization

For further structural understanding of the interaction of the DARPins with their target domains, we solved the X-ray crystal structure of both DARPins in complex with their respective target domains. The structure of DARPin 1800 in complex with the p73 OD revealed a 2:1 binding stoichiometry (couting the entire OD as one unit; Fig. 1F). Furthermore, the structure revealed that DARPin 1800 is binding to the hinge region of the OD which connects helix 1 with helix 2 [33, 34]. A detailed analysis of this interaction demonstrated that the interface of DARPin 1800 includes stretches of helix 1 and helix 2 forming multiple hydrophobic contacts as well as some hydrogen bonds (Supplementary Fig. S3 A, B). The high selectivity of DARPin 1800 towards the p73 OD, despite the high sequence identity with the other family members, can be explained by direct contact with non-conserved residues within the p73 OD including L380, P384 and L385.

We also solved a high-resolution crystal structure of DARPin B9 in complex with the p73 SAM domain, which showed a 1:1 stoichiometry, as is also indicated by the ITC measurements (Fig. 1F). The interaction interface is mainly located within helix 1 and helix 2 of the p73 SAM domain. Additional contacts are formed between some N-terminal residues as well as helix 5 of the p73 SAM domain and the DARPin (Supplementary Fig. S3C, D). The selectivity relative to the p63 SAM domain is mediated by hydrophobic contacts to a variety of non-conserved residues throughout the SAM domain, including P491, G499, P503, Y537 and T540.

### Dimerization of DARPins increases their functional affinity

**Fig. 2:**
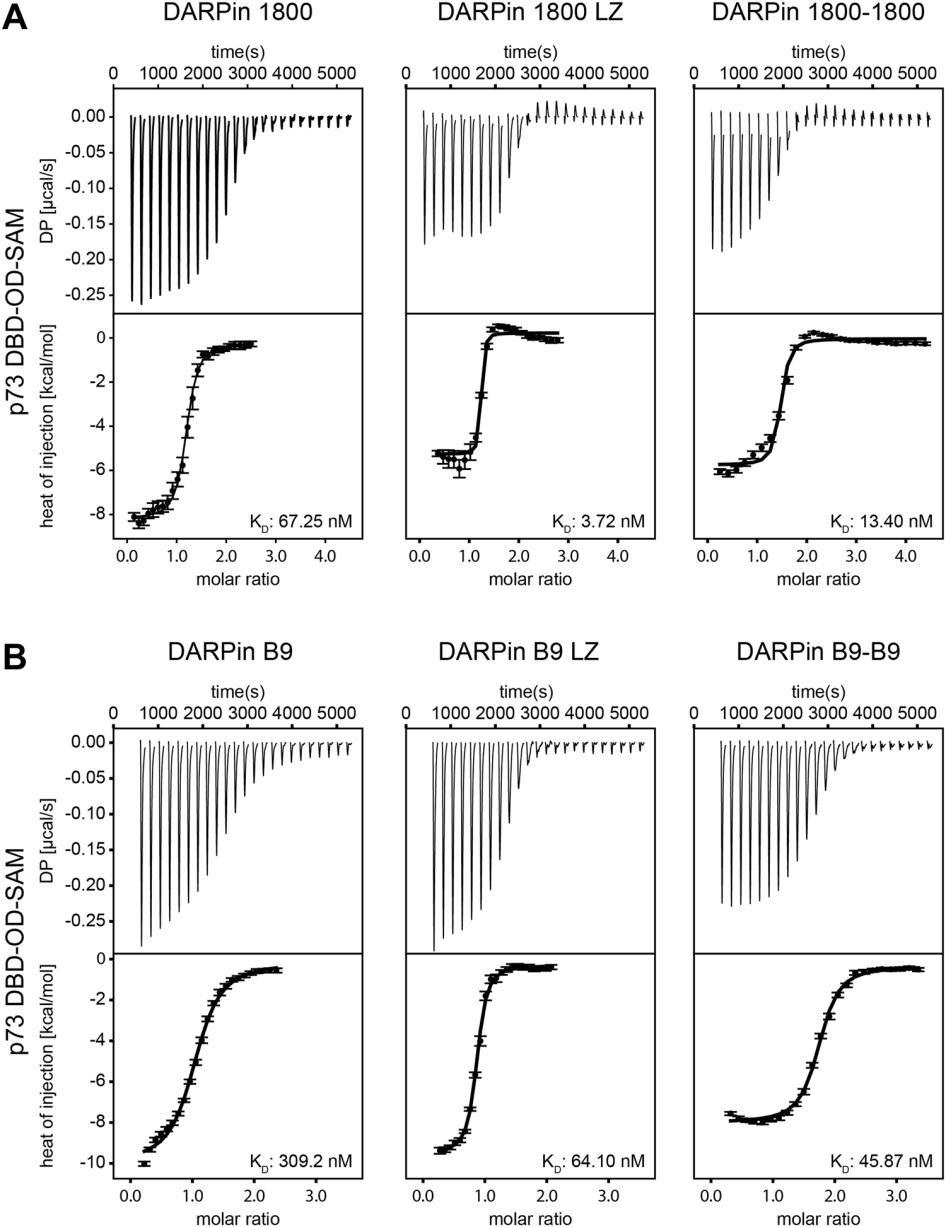
Dimerization of the selected DARPins increases their functional affinity. **A** Interaction study of p73 DBD-OD-SAM and DARPin 1800 (left panel), DARPin 1800 dimerized via a leucine zipper (DARPin 1800 LZ, middle panel) and DARPin 1800 fused to another DARPin 1800 via a (G_4_S)_4_ linker (DARPin 1800–1800, right panel) using ITC. The top diagram shows the raw measurement and the bottom diagram the integrated heat per titration step. The *K*_D_ value for the interaction is given in the bottom right corner. The measurement was performed at 25 °C. **B** Same experiment as in (**A**) but with DARPin B9, DARPin B9 LZ and DARPin B9-B9.

Previously, we have shown that the functional affinity of the DARPins to their target domain can be boosted by dimerizing the DARPin using a leucine zipper [29] as has also been shown for other DARPins before [35, 36]. We applied the same approach for the DARPins binding to p73 domains. Additionally, we dimerized the DARPins by creating linear fusion constructs using a (G_4_S)_4_ linker which was attached to the C-terminus of one DARPin and the N-terminus of the second DARPin. To investigate if the affinity of the DARPins towards p73 was increased, we performed ITC measurements with a p73 construct containing the DBD, the OD and the SAM domain (DBD-OD-SAM, amino acids 112–550 of TAp73α) and the respective DARPins. As a reference, the monomeric DARPins were included in the experiments. For DARPin 1800 we were able to increase the affinity from 67 nM of the monomeric DARPin to 4 nM for the DARPin dimerized via the leucine zipper domain (1800 LZ) and to 13 nM for the linear fusion construct 1800–1800 (Fig. 2A, Supplementary Table S2). In the case of DARPin B9 the affinity was increased from 309 nM (which is within this multi-domain construct significantly higher than the *K*_D_ measured for the isolated SAM domain) to 64 nM for the leucine zipper construct DARPin B9 LZ and to 46 nM for the linear fusion construct B9-B9 (Fig. 2B, Supplementary Table S2). Dimerized versions of the non-binding control DARPin did not show any binding to p73 DBD-OD-SAM (Supplementary Fig. S4).

### Immunofluorescence detection of p73 isoforms

**Fig. 3:**
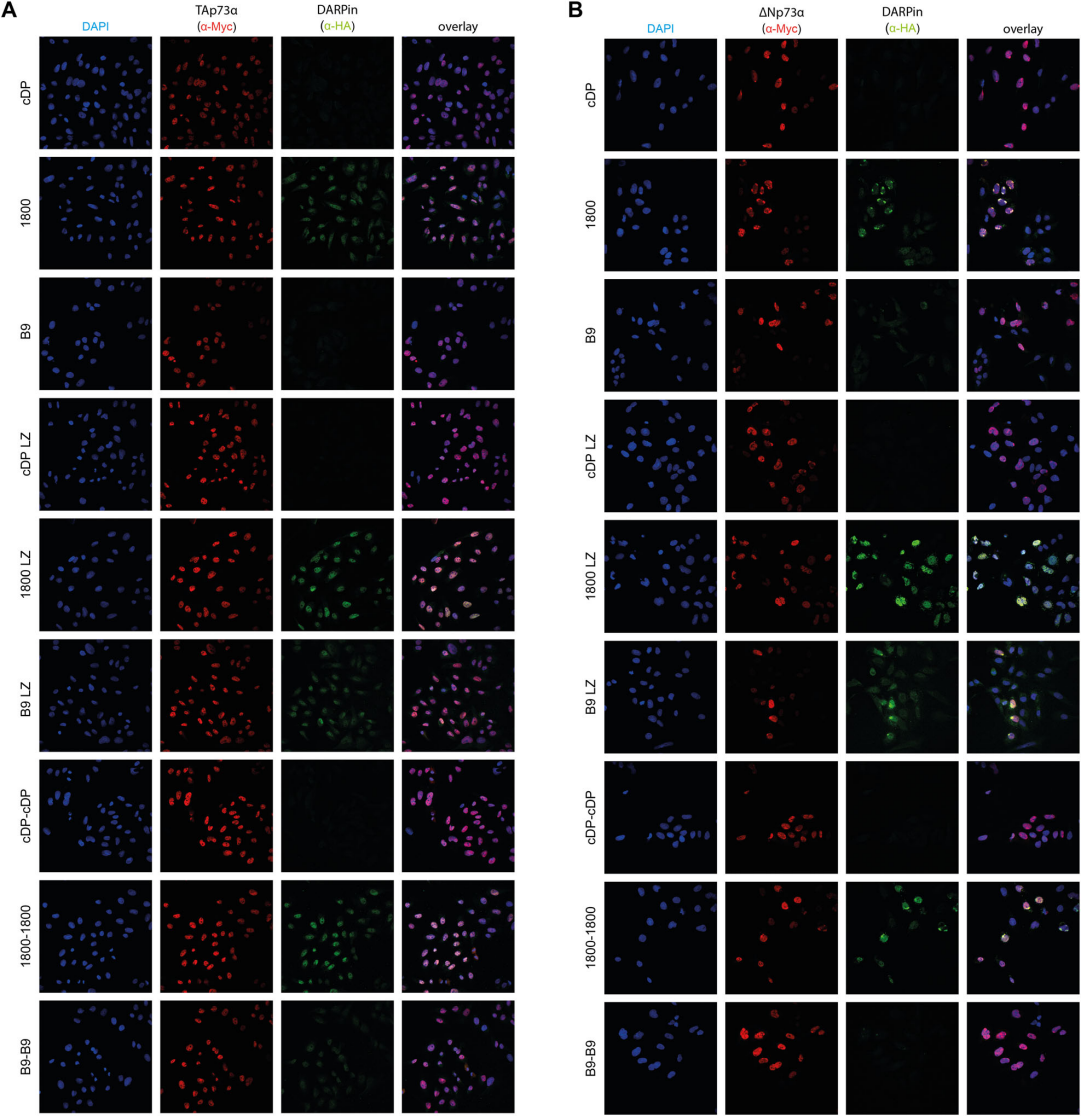
Detection of p73 isoforms in stable expressing U-2 OS cells. Cells expressing Myc-tagged TAp73α (**A**) or Myc-Tagged ∆Np73α (**B**) were fixed with formaldehyde and incubated with the indicated HA-tagged DARPin followed by incubation with goat anti-HA antibody (a190138a—Bethyl) and the secondary antibody Alexa Fluor 568 anti-goat (A11057—Life Technologies). The same cells were also incubated with mouse anti-myc antibody 4A6 (Millipore) and Alexa Fluor 647 anti-mouse antibody (A31571—Life Technologies) as both p73α isoforms were Myc-tagged. The control DARPin constructs do not show any signal above background.

The results so far demonstrated that the DARPins bind their domains with high selectivity and affinity and can be used as a tool for affinity precipitation experiments. Next, we wanted to develop the DARPins as a tool for the detection of p73 isoforms in cells. The DARPins were modified with an N-terminal HA-tag for this purpose. Stably expressing U-2 OS cell lines that either express Myc-tagged TAp73α, ∆Np73α or ∆Np63α were fixed using PFA-fixation and incubated with 100 nM DARPin solution overnight. Subsequently, the cells were stained for the Myc-tag of the p73 isoforms and the HA-tag of the DARPins. The results in Fig. 3A, B showed no HA-tag signal for the non-dimerized DARPin B9, while weak signals for the dimerized version B9 LZ are detectable for both TAp73α and ∆Np73α. The linear fusion construct B9-B9 showed a weak staining of TAp73α and no detectable staining of ∆Np73α. For DARPin 1800 a weak HA-tag signal is detectable for the monomeric DARPin, and a much stronger signal for the dimerized versions 1800 LZ and 1800–1800. All cells showing an HA-tag signal also stained for the anti-Myc antibody, indicating that the DARPins stained p73 with low background and high specificity. The control DARPin as well as the dimerized versions of the control DARPin did not show any HA-tag signals. To further validate the high specificity of the DARPins we performed staining with U-2 OS cells expressing ∆Np63α but no HA-tag signal was detectable for any of the DARPins again demonstrating the high specificity of the DARPins (Supplementary Fig. S5).

### Detection of p73 in mouse tissue lysates

**Fig. 4:**
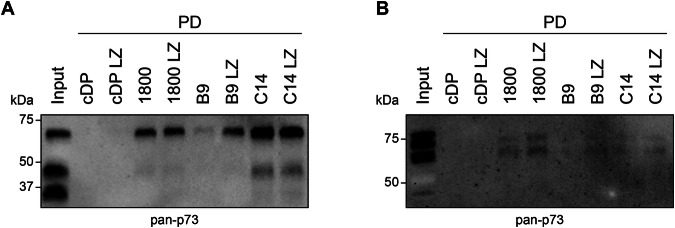
Detection of p73 in primary tissue. **A** Pulldown (PD) of p73 from mouse skin extract. The p73 OD-specific DARPin 1800 and its dimerized version 1800 LZ, the p73 SAM-specific DARPin B9 and its dimerized version B9 LZ, the DBD-specific DARPin C14 and its dimerized version C14 LZ as well as the respective control DARPin constructs were biotinylated and immobilized on Streptavidin magnetic beads followed by incubation with the mouse skin extract. Multiple bands corresponding to different isoforms of p73 were detected. However, it is not possible to identify the isoform based on the blot due to the similar molecular weight of several isoforms. The pulldown was analyzed by western blot with an anti-p73 antibody (ab 40658). All DARPins except the control DARPin constructs show pulldown signals. **B** Same experiment as in (a) but with mouse brain extract. Both experiments were done in biological triplicates with one representative replicate shown.

So far, all experiments were performed with purified proteins or in cell culture. To investigate whether our DARPins can also be used to detect p73 in primary tissue we performed pulldown experiments with lysates generated from mouse skin and mouse brain. We used mice as a source of primary tissue because human and mouse p73 have a very high sequence identity [37]. Homogenous extracts from mouse skin were incubated with biotinylated DARPins immobilized on streptavidin magnetic beads. Additionally, we included the previously described DARPin C14 which is known to bind to the DNA binding domain of p63 and p73 [29]. Furthermore, we included the dimeric version of all DARPins that have been generated by the addition of a leucine zipper (LZ) domain to the C-terminus of the DARPins. All DARPins were effective in pulldown experiments showing pulldown signals for p73 from mouse skin (Fig. 4A, Supplementary Fig. S1). However, only a relatively weak pulldown was observable for DARPin B9, while DARPin B9 LZ showed a pulldown efficiency comparable to the pulldown efficiencies of DARPins 1800 and C14. For DARPin 1800 and C14 no difference in pulldown efficiency was observable between the monomeric and the dimerized versions. We performed the same pulldown experiment with a homogenous extract derived from the mouse brain, but only very weak pulldown signals were observable for all DARPins (Fig. 4B, Supplementary Fig. S1). DARPin B9 and DARPin C14 showed nearly no pulldown of p73. Stronger pulldown signals were detectable for DARPin 1800, 1800 LZ, B9 LZ, and C14 LZ.

### DARPin-based bioPROTACs specifically degrade p73

**Fig. 5:**
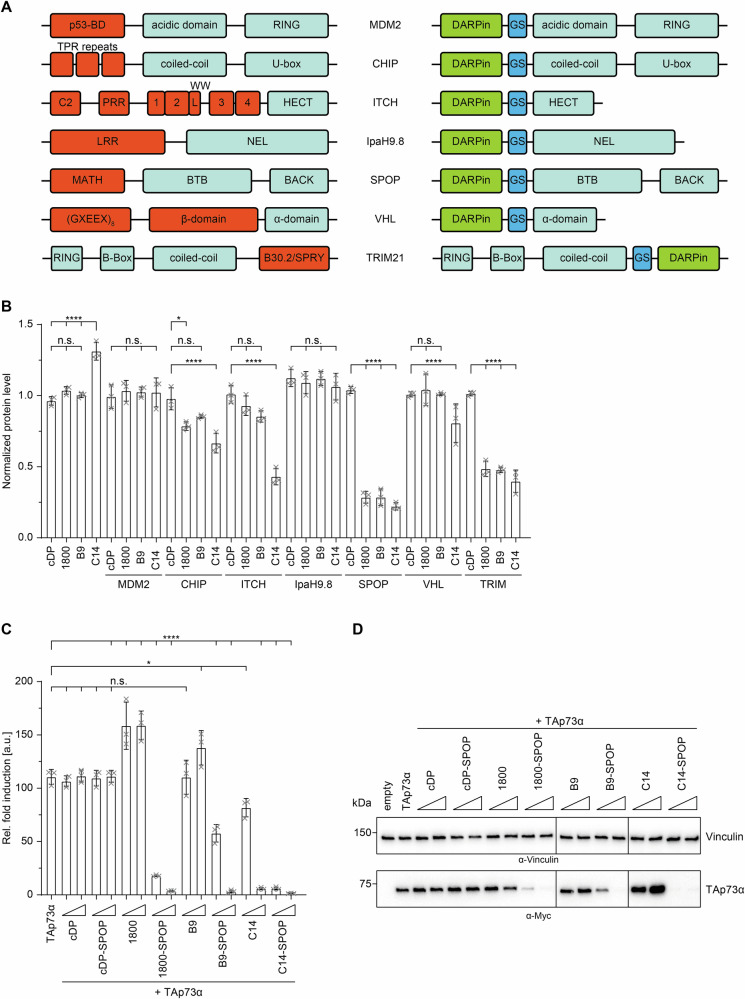
DARPin-based PROTACs degrade p73. **A** Schematic representation of the domain structure of the used E3-ligases (left). The natural substrate binding domain is colored in red. On the right side, a schematic representation of the DARPin-E3 chimera constructs is shown in which the substrate binding domain has been replaced by the DARPin (colored in green). **B** Screening of the efficiency of different DARPin-based PROTACs by a luciferase reporter assay using H1299 cells. The p73 OD-specific DARPin 1800, the p73 SAM-specific DARPin B9 and the DBD-specific DARPin C14 were fused to different E3-ligases or the E3-ligase adaptor protein SPOP. The resulting DARPin fusion constructs were co-transfected with HiBit-tagged TAp73α and the TAp73α protein level was determined 24 h after transfection using the HiBiT dual-luciferase reporter assay (Promega). For normalization, firefly luciferase was expressed from the same plasmid using an IRES. Fusion of the control DARPin to the respective E3-ligase served as negative control. Protein levels were determined by calculating the ratio between NanoLuc luminescence and Firefly luminescence and normalized to a sample not containing any DARPin or degrader. The assay was performed in biological triplicates. **C** Quantitative analysis of the transcriptional activity of TAp73α in the presence of the respective DARPin or DARPin-SPOP fusion. Triangles indicate increasing amounts of transfected DARPin or DARPin-SPOP plasmid DNA (5–25 ng plasmid DNA). The assay was performed in biological triplicates. The assay was performed in H1299 cells. **D** Western Blot of the experiment in (**C**). Myc-tagged p73 was detected using the anti-myc antibody 4A6 (millipore). Vinculin protein level was detected as a loading control using the anti-vinculin antibody 7F9 (Santa Cruz Biotechnology). Triangles indicate increasing amounts of transfected DARPin or DARPin-SPOP plasmid DNA (5–25 ng plasmid DNA). The bar diagrams in (**B**) and (**C**) represent mean values of three biological replicates and the error bars the respective standard deviations. Statistical significance was assessed by ordinary one‐way ANOVA (n.s.: *P* > 0.05, *P ≤ 0.05, **P ≤ 0.01, ***P ≤ 0.001, ****P ≤ 0.0001).

So far transcription factors like p73 have been classified as “difficult to drug” proteins as they lack binding pockets for small organic compounds that can be used as inhibitors. A recently developed alternative to classical inhibitor-based drug development is the Proteolysis Targeting Chimeras (PROTAC) approach in which a target protein is brought in close contact with an E3 ligase to get degraded. In the bioPROTACs approach, binding to the target protein is achieved by small protein-based binding modules that are directly linked to E3 ligases or E3 ligase adaptors [8–11]. With the DARPins as highly affine and selective binders, a very attractive class of binding modules for the development of bioPROTACs is available. We have developed DARPin-based bioPROTACs by replacing the substrate binding domain of several E3-ligases or E3-adaptor proteins with the respective DARPins. In particular, we replaced the substrate recognition domain of the E3-ligases MDM2, CHIP, ITCH, VHL, and TRIM, as well as the substrate binding domain of the cullin 3 adaptor SPOP and the bacterial E3-ligase mimic IpaH9.8 with our DARPins (Fig. 5A). In addition to the DARPins 1800 and B9 described in this study, we built the same constructs with DARPin C14 that has been previously identified as a high-affinity inhibitor of p63 and p73 [29]. DARPin C14 binds to the DNA binding interface of the DBD of p63 or p73 thereby blocking DNA binding and inhibiting transcription of p63 or p73 target genes.

Transcription factors such as the p53 family members are located in the nucleus and as a consequence, our degraders have to be located in the nucleus as well. We first examined the cellular localization of these bioPROTACs through transient transfection of H1299 cells followed by IF staining (Supplementary Fig. S6A–F). While certain DARPin-E3 chimeras were found in the nucleus (e.g. DARPin-MDM2 and DARPin-SPOP), others exhibited a more cytosolic localization (e.g. DARPin-Stub and DARPin-VHL). To attain nuclear localization for all bioPROTACs, we included the nuclear localization signal of SV40 (PKKKRKV) in all DARPin-E3 ligase chimeras.

Next, we employed the HiBit Dual Luciferase system (Promega) to assess the efficacy of the different DARPin-E3 fusion constructs for the degradation of the target proteins. For this purpose, we co-transfected H1299 cells with the DARPin-E3 fusion constructs and the pBit4.1-N[HiBit-IRES-luc2/CMV/Blast] vector in which TAp73α was introduced via restriction-dependent cloning using XhoI and XbaI. In this system a small part of the nanoLUC luciferase is N-terminally fused to the protein of interest, in this case TAp73α. After cell lysis the larger part of the nanoLUC luciferase is added for complementation together with substrate. For normalization, firefly luciferase is constitutively expressed from the same plasmid using an IRES. Twenty-four hours after transfection, firefly and nanoLuc luciferase activities were measured, and the relative protein levels were determined by calculating the ratio between both luciferase activities (Fig. 5B). As a reference, we co-transfected the respective DARPins with an N-terminal HA-tag and the SV40-NLS but without the E3 ligase fusion part.

The assay results revealed that none of the three DARPins induced degradation of TAp73α when fused to MDM2 or IpaH9.8. Significant degradation of TAp73α was observed for C14-CHIP, C14-ITCH and C14-VHL. However, the fusion of DARPin 1800 or B9 with these E3 ligases did not induce degradation of TAp73α. Among the tested bioPROTACs, the fusion of any of the DARPins with TRIM21 or SPOP demonstrated the most potent degradation, with the SPOP fusion constructs exhibiting a slightly higher efficiency. Consequently, the C14-SPOP, 1800-SPOP and B9-SPOP fusions were selected for further experiments. Notably, for all E3-ligases, the control DARPin-fusions did not induce any degradation of TAp73α.

We also explored the specificity of our bioPROTACs by co-transfecting the corresponding DARPin-SPOP fusion with the p63 isoforms TAp63α and ∆Np63α. Once again, the HA-tagged DARPins served as a reference (Supplementary Fig. S7A, B). This assay demonstrated the high specificity of the 1800-SPOP and B9-SPOP fusions, as no significant degradation of either p63 isoform was observed. DARPin C14, which is known to bind to p63 and p73, efficiently degraded both p63 isoforms when fused with SPOP. The control DARPin-SPOP fusion, utilized as a negative control, demonstrated no degradation of p63. To explore whether the degradation efficiency relies on the transfected quantity of degrader DNA and to identify the optimal DNA amount, we co-transfected increasing amounts of the respective DARPin-SPOP fusion with pBit4.1-TAp73α. The degradation efficiency was calculated by assessing the p73 protein level through the dual luciferase assay (Supplementary Fig. S7C). This assay revealed that degradation efficiency is indeed dependent on the transfected bioPROTAC amount, reaching maximum efficiency at approximately 5–10 ng of transfected DNA. As observed in prior assays, the control DARPin-SPOP fusion exhibited no effect.

To further explore the degradation of TAp73α mediated by DARPin-E3 constructs and its impact on the transcriptional activity of p73, we conducted a transactivation assay using firefly luciferase under the control of the pBDS-2 promotor, which consists of three copies of the p53 binding site from the 14-3-3σ promotor, as reporter. Notably, DARPins 1800 and B9 enhanced p73 activity when co-transfected with TAp73α, while the control DARPin showed no such effect (Fig. 5C). However, this increased activity did not correspond to an elevation in protein levels, as observed in the corresponding Western Blots (Fig. 5D). As previously documented, DARPin C14 inhibits TAp73α in a concentration-dependent manner [29]. When using the C14-SPOP fusion, this inhibition is more efficient than DARPin C14 itself. This is due to a combined inhibition by binding of the DARPin to the DNA binding interface and degradation. with TAp73α being undetectable on the Western blot even at the lowest applied concentration of C14-SPOP (Fig. 5C, D, Supplementary Fig. S1). The other two DARPin-SPOP fusion constructs inhibited TAp73α as well through degradation, albeit with a lower efficiency as compared to the C14-SPOP fusion (Fig. 5C).

### DARPin-based bioPROTACs reverse promotor squelching by ∆Np73α

**Fig. 6:**
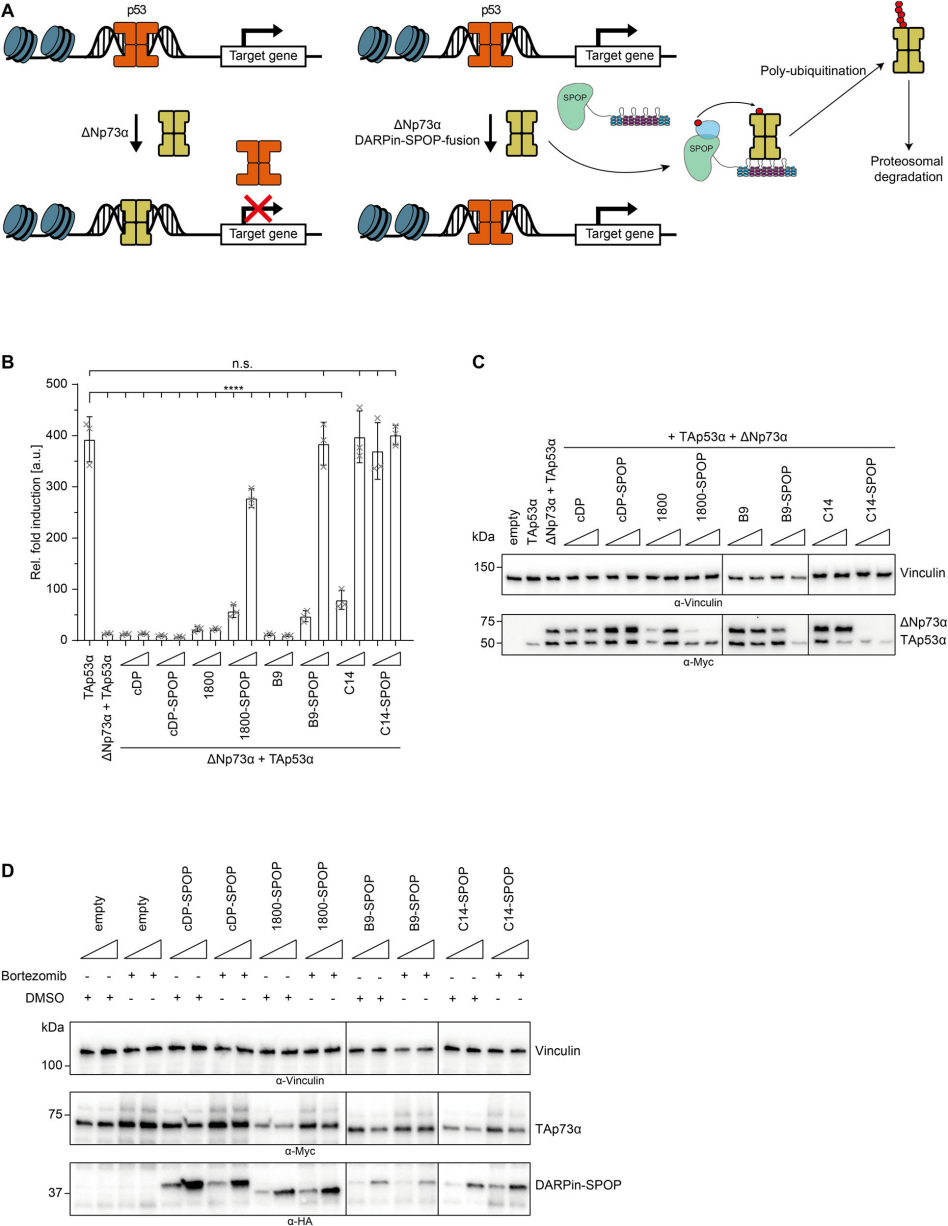
DARPin-SPOP fusions restore transcriptional activity of p53 in presence of ΔNp73α and the DARPin-SPOP induced degradation of TAp73α is dependent on the proteosomal pathway. **A** Schematic representation of the inhibitory effect of ΔNp73α on the transcriptional activity of p53. High concentrations of ΔNp73α (yellow) displace p53 (orange) from the promoter sequences (left). Degradation of ΔNp73α by DARPin-SPOP fusions enables binding of promotor sequences and thus transcription of the affected genes (right). (adapted from [29]). **B** Quantitative analysis of the transcriptional activity of Myc-tagged p53 in the presence of Myc-tagged ΔNp73α with or without the respective DARPin-SPOP fusion. Degradation of ΔNp73α by DARPin-SPOP fusions restores p53 transcriptional activity. The assay was performed in H1299 cells. The bar diagrams show the mean values and error bars of the corresponding SD of three biological replicates. Statistical significance was assessed by ordinary one-way ANOVA (n.s.: *P* > 0.05, *P ≤ 0.05, **P ≤ 0.01, ***P ≤ 0.001, ****P ≤ 0.0001). **C** Western Blot of the experiment in (B). Myc-tagged p53 and Myc-tagged ΔNp73α were detected using the anti-myc antibody 4A6 (millipore). Vinculin was detected as a loading control using the anti-vinculin antibody 7F9 (Santa Cruz Biotechnology). Triangles indicate increasing amounts of transfected DARPin or DARPin-SPOP plasmid DNA (5–25 ng plasmid DNA). **D** Proteasome inhibition assay to investigate if the degradation is dependent on the proteosomal pathway. U-2 OS Flp-In/T-REx cells stably expressing Myc-tagged TAp73α were transfected with 5 ng or 25 ng DNA of selected HA-tagged degrader (1800-SPOP, B9-SPOP and C14-SPOP). cDP-SPOP was used as negative control. 12 h after transfection, cells were treated with DMSO or the proteasome inhibitor bortezomib. TAp73α degradation with and without proteasome inhibitor was determined by SDS-PAGE followed by western blot with the anti-myc antibody 4A6 (millipore). The degraders were detected using the anti-HA antibody 16B12 (Biolegend), vinculin protein level was detected as a loading control using the anti-vinculin antibody 7F9 (Santa Cruz Biotechnology). The experiment was performed in biological triplicates with one representative replicate shown.

It is well known that p73 isoforms that lack the TA domain such as ∆Np73α are transcriptionally inactive and can act as dominant negative inhibitors of transcriptionally active p53 family members [38]. Moreover, there are reports suggesting an upregulation of these isoforms in certain cancers, leading to the inhibition of p53 and TAp73α [39]. The DARPin-E3 chimeras could represent an innovative tool to counteract the inhibitory effect of ∆Np73α on p53, as depicted in Fig. 6A. To test this hypothesis, we co-transfected Myc-tagged p53 with Myc-tagged ∆Np73α and the DARPin or the corresponding DARPin-SPOP fusion, conducting a luciferase reporter assay to assess p53 activity. Co-transfection of Myc-tagged p53 with Myc-tagged ∆Np73α resulted in a nearly complete depletion of p53 activity (Fig. 6B). However, co-transfection of p53, ∆Np73α and the 1800-SPOP fusion restored p53 activity. The Western Blot shown in Fig. 6C indicated that this reactivation was based on the degradation of ∆Np73α. A similar effect was observed for the B9-SPOP fusion. The inhibitory DARPin C14 alone was sufficient to reactivate p53 because this DARPin prevents binding of ΔNp73α to DNA. However, the assay revealed that the C14-SPOP fusion was more efficient than DARPin C14 alone, as p53 activity was completely restored even at low concentrations. Compared to the 1800-SPOP and the B9-SPOP fusion, the efficacy of C14-SPOP was higher as well. Interestingly, the DARPin 1800 seems to have a stabilizing effect on ΔNp73α as the transfection of this DARPin resulted in a higher cellular concentration of ΔNp73α (Fig. 6C, Supplementary Fig. S1). Presumably this effect is due to stabilizing the tetrameric state of the p73, making its proteasomal degradation by endogenous ligases less efficient.

### Degradation of p73 by DARPin-based bioPROTACs is dependent on the proteasome

To demonstrate that the DARPin-E3-induced degradation of TAp73α relies on the proteasomal pathway and does not occur through alternative pathways, we transfected a cell line stably expressing Myc-tagged TAp73α with the corresponding DARPin-E3 fusion. Subsequently, we treated the cells with either DMSO as a negative control or the proteasome inhibitor bortezomib (Fig. 6D, Supplementary Fig. S1). The Western Blot analysis showed that in case of the SPOP-fusions an increased p73 protein level was observed in samples treated with bortezomib demonstrating, that the degradation of p73 is indeed dependent on the proteasome.

## Discussion

The development of the Targeted Protein Degradation approach has provided a conceptually new strategy for the treatment of diseases by degrading the disease-relevant proteins. One big advantage is that molecular glues or bi-functional degraders do not have to bind to a biologically functional site like the active site of enzymes but can bind anywhere on the target protein provided this binding brings lysine residues of the target protein in close proximity to the E3 ligase for productive ubiquitination. This interaction can also be weaker than the interaction of classical inhibitors with their targets as the interaction only needs to be transient for the ubiquitination reaction. Several ongoing clinical trials show that the TPD strategy is working and mey become a very valuable addition to classical inhibitors.

This approach can in principle be extended to virtually all proteins if the target protein recognition module is replaced with a small protein-based binder and directly fused to an E3 ligase. These bioPROTACs containing nanobodies, monobodies, DARPins or natural ligands have been tested successfully [8–11]. Here we show that the DARPins that we have developed as highly selective binders for p73 fused to E3 ligases can be used to degrade either all p73 isoforms or selected ones. For multi-domain proteins such as p73, DARPins can be developed targeting each folded domain and the most effective one to induce degradation can be selected. Our tests so far have demonstrated that the fusion to the adaptor protein SPOP for the Cullin-RING-based BCR (BTB-CUL3-RBX1) E3-ligase complex seems most efficient in this context. This adaptor protein has also previously been shown to be highly effective in the context of other bioPROTACs [8] screens.

The advantages of the high selectivity of the DARPins and their comparably straightforward development via well established in vitro selection procedures make the design of DARPin-E3 ligase-based bioPROTACs in principle possible for all proteins that contain at least one folded domain. These selective degraders can be used as a research tool to investigate the physiological consequences of degrading a specific protein. While the siRNA approach is also very effective in removing a protein from a cell, the DARPin-based PROTACs approach has the advantage that it can be selective for certain conformations, oligomeric states or mutant form of a protein without degrading other conformations or the wild-type protein. One further advantage of bioPROTACs compared to siRNA is that they remove also proteins effectively that have a long half-life in vivo. The use of siRNA will shut down the translation of new proteins but the already existing population could still be present for longer periods of time [8].

An example of a conformation-selective DARPin is our previously described DARPin 8F1 which binds the tetrameric state of the activated form of TAp63α but not its dimeric inactive form [29]. TAp63α is highly expressed in oocytes where it serves as a surveillance factor for the genetic integrity with the active tetrameric form inducing apoptosis [40, 41]. With a DARPin 8F1 based bioPROTAC, induction of apoptosis might be suppressed without targeting the inactive dimeric state of the protein. Another example for the use of conformation-specific DARPins are p53 mutants which in most cases differs from wild type p53 by a single nucleotide exchange which makes it very difficult to remove only the mutated form using siRNA. As p53 mutants adopt conformations that differ from the wild-type structure, selective DARPins might be used to target only the mutant form. Selective DARPins have been developed against the extracellular signal-regulated kinase (ERK) both in its nonphosphorylated (inactive) or doubly phosphorylated (active, p-ERK) form [42] and the c-Jun N-terminal kinase-1 and 2 (JNK1 and JNK2) [43]. Again, a combination of these highly specific DARPins with an E3 ligase makes it possible to create bi-functional phosphorylation-state specific degraders.

The potential therapeutic use of DARPin-based bioPROTACs in human patients is complicated by the need to get a protein into the cellular environment. One strategy for translocating the protein has been to engineer it to bind cationic and ionizable lipids via electrostatic interactions for cytosolic delivery [44]. Another option is to deliver the genetic material instead, either via viral or non-viral strategies. The mRNA/lipid nanoparticle method offers a new way to achieve this goal. Clearly, the conditions for producing a vaccine and using the method to deliver a therapeutically active protein to cells are very different [24]. In the second case the concentration must be much higher and as many as possible cells must take up the particles and produce the protein. In addition, for a therapeutic application long time treatments might be necessary. Very recently, the intermediate results of the first phase I/II clinical trial with human patients in which the mRNA/lipid nanoparticle method was used to replace a dysfunctional enzyme in the liver have been published [28]. These results have demonstrated that treatment of human patients for up to a year with these mRNA/lipid nanoparticles at the used concentrations is safe.

Nanoparticles of most formaulations are known to accumulate in the liver, making the liver an excellent target for any lipid nanoparticle based therapy, based on mRNA or protein. The recent development of lipids that can target lipid nanoparticles also to lung or spleen and the detection of the underlying mechanisms have opened the door for the delivery of mRNA via lipid nanoparticles specifically to different organs.

## Methods

### Protein expression and purification

DARPins and p53 family domains were expressed in *E. coli* and purified as described before [29, 30]. In brief, *E. coli* BL21(DE3) Rosetta cells were transformed with the respective pET-15b expression plasmid. Cells were grown in 2xYT medium until an optical density of 0.8 was reached. Protein expression was induced with 0.6 mM IPTG for 16 h at 16 °C. Cells were harvested by centrifugation, resuspended in IMAC A buffer (50 mM Tris pH 8, 400 mM NaCl, 20 mM β-mercaptoethanol) supplemented with RNAse (Sigma), DNAse (Sigma), lysozyme (Sigma), and self-made protease inhibitors, and were lysed by sonification. After clearing the lysate by centrifugation, the supernatant was supplemented with 30 mM imidazole and loaded on a pre-equilibrated immobilized metal ion affinity chromatography (IMAC) column (HiTrap IMAC Sepharose FF, Cytiva) following an IMAC purification protocol. The eluted protein was simultaneously dialyzed to IMAC A buffer and digested with TEV protease (home-made). TEV protease and undigested protein were separated by a reverse IMAC step. The purified proteins were further purified and buffer exchanged by size-exclusion chromatography (SEC) with SEC buffer (50 mM Tris, pH 8, 150 mM NaCl, 0.5 mM TCEP) using a Superdex 75 10/300 column (Cytiva) using an ÄKTA purifier system at 4 °C. Central peak fractions were collected, concentrated to a concentration 300 to 500 µM (Amicon Ultra Centrifugal Filters, Millipore) and flash-frozen in liquid nitrogen prior to storage at -80 °C until use. The purity and molecular weight of purified proteins were monitored by SDS-PAGE and LC-ESI-TOF-mass spectrometry.

### DARPin biotinylation

Avi-tagged target proteins for DARPin selections and DARPins containing an N-terminal Avi-tag were enzymatically biotinylated in vitro using the *E. coli* biotin ligase BirA. BirA was subcloned into a pET-15b-GFP-His8-TEV *E. coli* expression vector (Novagen, Merck KGaA) and was expressed and purified as described before [29]. The in vitro biotinylation was performed by mixing GFP-BirA in a 1:50 molar ratio with the respective Avi-tagged DARPin in SEC buffer supplemented with 10 mM ATP, 10 mM MgCl_2_, 0.5 mM biotin. The mixture was incubated for 16 h at 16 °C and subsequently separated SEC using a Superdex 75 10/300 column on an ÄKTA Purifier system (GE Healthcare). Biotinylated DARPin fractions were pooled and analyzed by LC-ESI-TOF-mass spectrometry. Only DARPins showing close to 100% labeling efficacy were used for experiments.

### Selection and screening of DARPin binders

To generate DARPin binders specific for p73, either biotinylated p73 OD (aa 351–398 of TAp73α) or the p73 SAM domain (aa 489–550 of TAp73α) was immobilized on either MyOne T1 streptavidin-coated beads (SA; Pierce) or Sera-Mag neutravidin-coated beads (NA, GE), depending on the particular selection round, and these beads were alternated. Ribosome display selections were performed essentially as described [19, 20], but using a semi-automatic KingFisher Flex MTP96 well platform.

The library includes N3C-DARPins with the original randomization strategy as reported [31] but uses a stabilized C-cap [16, 45, 46]. Additionally, the library is a mixture of DARPins with randomized and non-randomized N- and C- terminal caps, respectively [16, 47]. Successively enriched pools were cloned as intermediates in a ribosome display-specific vector [47]. Selections were performed over four rounds with decreasing target concentration and increasing washing steps to enrich for binders with high affinities. In addition, a prepanning with BSA blocked SA or NA beads was performed to eliminate unspecific DARPins in rounds two to four in the selection for p73 OD.

The final enriched pool was cloned as fusion construct into a bacterial pQE30 derivative vector with a N-terminal MRGS(H)8 tag and C-terminal FLAG tag via unique BamHI x HindIII sites containing a *T5lac* promoter and *lacIq* for expression control. After the transformation of *E. coli* XL1-blue, 380 single DARPin clones were expressed and tested for target binding in a Homogeneous Time Resolved Fluorescence (HTRF)- or ELISA-based assay From the identified binders, 32 random target-specific clones for both targets were sequenced and single clones identified. For the selection of DARPins against the p73 SAM domain (aa 489–550) DARPins, 21 DARPins were single clones and unique. Out of this panel, clone B9 described in this study was derived. For the selection of DARPins against the p73 OD DARPins, clone 1800 described in this study was obtained only after monoclonalization of a double clone.

The final enriched pool was cloned as fusion construct into a bacterial pQE30 derivative vector with a N-terminal MRGS(H)8 tag and C-terminal FLAG tag via unique BamHI x HindIII sites containing a *T5lac* promoter and *lacIq* for expression control.

After the transformation of *E. coli* XL1-blue, 380 single DARPin clones for p73 OD or the p73 SAM domain (aa 489–550) were expressed in 1‑mL scale in deep-well plates by addition of IPTG (isopropyl β-D-1-thiogalactopyranoside). Cells were harvested by centrifugation, and lysed with the addition of B-Per Direct detergent plus Lysozyme and Nuclease (Pierce). The lysates were cleared by centrifugation. These bacterial crude extracts of single DARPin clones were subsequently used in a Homogeneous Time Resolved Fluorescence (HTRF)-based screen for target p73 OD, while for target p73 SAM domain (aa 489–550) an ELISA-based assay was used to identify potential binders. In HTRF, binding of the FLAG-tagged DARPins to streptavidin-immobilized biotinylated p73 OD was measured using FRET (donor: Streptavidin-Tb cryptate (610SATLB, Cisbio), acceptor: mAb anti-FLAG M2-d2 (61FG2DLB, Cisbio). Further HTRF measurement against ‘No Target’ allowed for discrimination of p73 (aa 112–594))-specific hits. Experiments were performed at room temperature in white 384-well Optiplate plates (PerkinElmer) using the Taglite assay buffer (Cisbio) at a final volume of 20 μl per well. FRET signals were recorded after an incubation time of 30 min using a Varioskan LUX Multimode Microplate Reader (Thermo Scientific). HTRF ratios were obtained by dividing the acceptor signal (665 nm) by the donor signal (620 nm) and multiplying this value by 10,000 to derive the 665/620 ratio. The background signal was determined by using reagents in the absence of DARPins. In ELISA, binding of the FLAG-tagged DARPins to neutravidin-immobilized biotinylated p73 SAM domain (aa 489–550) was detected using a mouse monoclonal anti-FLAG-M2 antibody (Sigma, F3165) and a goat-anti-mouse antibody coupled to alkaline phosphatase as secondary antibody (Sigma, A3562). Target-specific binding of DARPins was analyzed by following the hydrolysis of para-nitrophenylphosphate at 405 nm in an ELISA-plate reader (Tecan). The background signal was determined by using reagents in the absence of biotinylated target protein.

From the identified binders, 32 random target-specific clones for both targets were sequenced and single clones identified. For the selection of DARPins against the p73 SAM domain (aa 489–550) DARPins, 21 DARPins were single clones and unique. These DARPins were characterized regarding their specificity towards p73 by pulldown assays with isoforms of all p53 family members yielding DARPin 1800 as a specific binder for the p73 OD and DARPin B9 as a specific binder for the p73 SAM domain.

### Molecular cloning

For recombinant protein expression of DARPins and all p63/p73 constructs, derivatives of a pET15b plasmid (Novagen, Merck KGaA) were used. Inserts were generated by PCR and introduced into pET15b-His_10_-TEV, pET15b-His_10_-TEV-Avi or pET15b-His_10_-TEV-HA (N-terminal His_10_-tag followed by a tobacco etch virus (TEV) protease cleavage site (and Avi- or HA-tag)) by subcloning using BamHI and XhoI restriction sites. For transient expression in mammalian cell culture, PCR-generated inserts were introduced in the pcDNA3.1(+) Myc plasmid (Invitrogen, Thermo Fisher Scientific Inc) by subcloning using BamHI and XhoI resctriction sites. For the generation of stable cell lines, PCR-generated inserts of p63 or p73 isoforms were introduced in a pcDNA5 FRT/TO plasmid by subcloning using BamHI and XhoI resctriction sites.

For generation of the leucine zipper DARPin constructs the protein coding sequence including the DARPins C-terminally fused to a (G_4_S)_4_-linker followed by the leucine zipper sequence (amino acids 250–281 of yeast GCN4) was synthesized (Eurofins). This gene strand was inserted into a pET15b-His_10_-TEV, pET15b-His_10_-TEV-Avi or pET15b-His_10_-TEV-HA by subcloning using BamHI and XbaI resctriction sites.

For generation of DARPin-E3 chimeras, a PCR generated insert of the respective E3 ligase was introduced into the pcDNA3.1 (+) HA plasmid (Invitrogen, Thermo Fisher Scientific Inc) using XhoI and XbaI. Subsequently, the respective DARPins were introduced by subcloning with BamHI and XhoI.

### Cell culture

The non-small cell lung cancer cell line H1299 (ATCC, CRL5803) was cultured in RPMI medium 1640 (Gibco), containing 10% FBS (Capricorn Scientific), 100 U/ml penicillin (Gibco) and 100 μg/ml streptomycin (Gibco) at 37 °C and 5% CO_2_. For recombinant protein expression, the H1299 culturing medium was exchanged with antibiotic-free medium, and cells were transfected using Lipofectamine 2000 (Thermo Fisher Scientific) according to the manufacturer’s instructions. Six hours after transfection the medium was exchanged to standard H1299 culturing medium.

For a generation of stable cell lines inducible expressing TAp73α, ∆Np73α or ∆Np63α, the Flp-In T-REx system (Thermo Fisher Scientific) was used. The T-REx-U-2 OS cell line was cultured for two weeks in DMEM medium (Gibco), containing 10% FBS (Capricorn Scientific), 4 μg/ml blasticidin (Gibco), 333 μg/ml Zeocin (Gibco), 100 U/ml penicillin (Gibco), 100 μg/ml streptomycin (Gibco) and 1 mM pyruvate (Gibco) at 37 °C and 5% CO_2_. T-REx-U-2 OS cells were transferred into six-well plates for transfection using Lipofectamin 2000 transfection reagent (Thermo Fisher scientific) and were transfected with pcDNA5/FRT/TO plasmid (Thermo Fisher Scientific) containing either TAp73α, ∆Np73α or ∆Np63α, and the pOG44 plasmid (Thermo Fisher Scientific) expressing the Flp recombinase according to the manufacturer’s instructions. After transfection, cells were cultured in DMEM containing 10% tetracycline-free FBS (Bio Cell) for 48 h and re-seeded into 15 cm dishes. Twenty-four hours after re-seeding, the medium was exchanged to selection medium (DMEM containing 10% tetracycline-free FBS, 4 μg/ml blasticidin, 200 μg/ml hygromycin (Thermo Fisher Scientific), 100 U/ml penicillin, 100 μg/ml streptomycin and 1 mM pyruvate) and cells were cultured until a non-transfected control showed no viable cells (about 10–14 days). For monoclone selection, twelve single colonies of each cell line were isolated and expression of target proteins was induced by the addition of 1 µg/mL tetracycline (Thermo Fisher Scientific) to the selection medium. After twenty-four hours, the expression of target proteins was analyzed by Western Blot. The best three monoclones were selected for further experiments.

The H1299 cell line was obtained from ATCC. The T-REx-U-2 OS cell line was a gift from Christian Behrends (Ludwig-Maximilians-University (LMU), Munich, Germany). All cell lines used in this study were routinely tested for mycoplasma contaminations.

### Gel electrophoresis and western blotting

Purified proteins were combined with SDS loading buffer (containing 250 mM Tris, pH 8.0, 7.5% (w/v) SDS, 25% (w/v) glycerol, 12.5% (v/v) β-mercaptoethanol, and 0.025% (w/v) bromophenol blue), subjected to denaturation at 95 °C, and then separated on manually prepared 4–16% Tris-glycine gels. The gels were subsequently stained using Quick Coomassie Stain (NeoBiotech) following the manufacturer’s guidelines.

For immunoblotting, samples were either mixed with SDS loading buffer or NuPAGE LDS buffer (Thermo Fisher Scientific) supplemented with DTT, denatured at 95 °C, and applied onto 4–15% Mini-PROTEAN TGX Stain-Free Precast Protein gels (Bio-Rad). Gel transfer was carried out using the TransBlot Turbo Transfer System (Bio-Rad) as per the manufacturer’s instructions. Membranes were blocked for 1 h in a blocking buffer (TBS, 0.05% (v/v) Tween-20, 5% skim milk powder, Sigma-Aldrich) and then incubated with the primary antibody in a blocking buffer overnight with shaking at 4°C. Following three washes with TBS-T, membranes were incubated with the secondary antibody in a blocking buffer under shaking conditions for 1 h at room temperature. Subsequently, membranes underwent three additional washes with TBS-T and were analyzed by adding Amersham ECL Prime WB Detection Reagent (Cytiva). Western blot signal quantification was performed using ImageLab (version 6.1, Bio-Rad).

The following antibodies and dilutions were used: anti-myc (1:2000, clone 4A6, Millipore), anti-p73 (1:2000, ab40658, Abcam), anti-HA (1:1000, clone 16B12, BioLegend) anti-vinculin (1:2000, clone 7F9, Santa Cruz Biotechnology), goat anti-mouse HRP (1:5000, A9917, Sigma Aldrich), goat anti-rabbit HRP (1:5000, 111-035-144, Jackson ImmunoResearch).

### Pulldown assays

Recombinant target proteins (cloned into a pcDNA3 vector) were expressed in H1299 cells. Endogenous target proteins were pulled down from tissues samples of eight-day-old (P8) female CD-1 mice, purchased from Charles River Laboratories. Cell and tissue lysates for pulldown samples were generated as described before [29]. Magnetic Dynabeads MyOne Streptavidin T1 (Thermo Fisher Scientific) were pre-incubated with an excess of biotinylated DARPin in Pulldown (PD) wash buffer (50 mM Tris, pH 8, 150 mM NaCl, 0.1% (v/v) Tween-20) while rotating for 1 h at 4 °C. Subsequently, the beads were washed three times with PD wash buffer and were resuspended in the same volume of PD wash buffer as before to maintain magnetic beads concentrations. For PD experiments, 10 µl DARPin loaded beads were mixed with freshly prepared cell lysate or tissue lysate and the total volume was adjusted to 1000 µl with pD wash buffer supplemented with 1x Complete Protease inhibitor (Roche). The PD samples were incubated rotating overnight at 4 °C. The beads were washed five times with 1000 µl PD wash buffer and bound proteins were eluted by boiling in 50 µl LDS buffer at 70 °C for 10 min. Samples were analyzed by Western Blot. Myc-tagged recombinant target proteins were detected using a mouse anti-myc antibody (1:2000, clone 4A6, Millipore). Endogenous p73 was detected using a rabbit anti-p73 antibody (1:1000, ab40658, Abcam).

### Isothermal titration calorimetry

All titration experiments were performed using a MicroCal VP-ITC microcalorimeter (Malvern Instruments Ltd, UK). DARPins and target proteins were dialyzed against ITC buffer (50 mM HEPES, pH 7.4, 150 mM NaCl, 0.5 mM TCEP). Target proteins were titrated to constant concentrations of DARPin in 10 μl steps with total injections of 25 and 250 s spacing time at indicated temperatures. The reference power was set to 25 μCal/s and stirring speed to 307 rpm. Automated unbiased baseline calculation and curve integration was done with NITPIC [48]. Thermodynamic parameters and the equilibrium constant were calculated by SEDPHAT [49]. The first data point was always excluded from the analysis.

### Protein crystallization and structure determination

Protein complexes consisting of DARPin and the respective p73 domain were prepared by mixing the purified proteins in SEC buffer. In case of the p73 SAM complex, proteins were mixed in 1:1 molar ratio. For the p73 OD complex, a 1:2 molar ratio (OD:DARPin) was used. After overnight incubation at 16 °C, unbound proteins were separated from the complex by SEC in crystallization buffer (20 mM Tris pH 7.8, 50 mM NaCl, 0.5 mM TCEP) using a Superdex 75 10/300 column. The central peak fractions corresponding to the complex were pooled and concentrated to a final complex concentration of ~300 µM. Protein purity was assessed by SDS-PAGE and LC-ESI-TOF-MS. Crystals were grown at 20 °C using the sitting drop vapor diffusion and the condition containing 30% PEG2000MME and 0.15 M potassium bromide. Crystals were cryo-protected with mother liquor containing 22% glycerol prior to flash-freezing in liquid nitrogen. Diffraction data were collected at the Swiss light source (Villigen, Switzerland). The obtained diffraction data were integraded with the program XDS^42^ and scaled with AIMLESS^43^. The crystal structures of the complexes were solved by molecular replacement using Phaser^44^ with published structures of the p73 OD (PDB: 5HOB) and DARPin (PDB: 6FP7) as search models. In the case of the p73 SAM domain-DARPin B9 structure a single DARPin (PDB: 6FP7) was used as a search model. Model building and refinement was performed using COOT ^45^ and REFMAC5^46^. X-ray data collection and refinement statistics are listed in Supplementary Table S3. Structural figures in this paper were prepared using PyMol (pymol.org).

### Immunofluorescence staining

Stable T-REx-U-2 OS cell lines expressing TAp73α, ∆Np73α or ∆Np63α were seeded on cover slips (Carl Roth). Expression of target genes was induced by addition of 1 µg/mL tetracycline (Thermo Fisher Scientific) to the culture medium. Twenty-four hours after induction of protein expression, cells were washed twice with PBS and fixed with Roti Histofix 4% (Carl Roth) for 10 min at room temperature. Fixed cells were washed twice with PBS, followed by permeabilization with PBS-T (PBS supplemented with 0.1% Triton X-100, Carl-Roth) for two times 5 min at room temperature. Permeabilized cells were blocked in blocking buffer (PBS-T supplemented with 1% BSA, Carl Roth) for 20 min at room temperature. Afterwards, cells were incubated with 100 nM HA-tagged DARPin or HA-tagged Leucine Zipper DARPin and mouse anti-myc antibody (1:500, clone 4A6, Millipore) in blocking buffer overnight at 4 °C. Cells were washed five times with PBS-T and incubated with Alexa Fluor 568 anti-goat antibody (1:200, A11057, Life Technologies) and Alexa Fluor 647 anti-mouse antibody (1:200, A31571, Life Technologies) in blocking buffer for 2 h at room temperature. Slides were washed five times with PBS-T and coverslips were mounted using Mowiol (Carl Roth) mounting medium which was supplemented with DAPI (Thermo Fisher Scientific). A detailed recipe of the mounting medium can be found at CSH protocols (http://cshprotocols.cshlp.org/content/2006/1/pdb. rec10255). The slides were dried several days before imaging with a LSM 780 confocal laser scanning microscope (Zeiss).

### Protein degradation assay

To investigate the degradation of target proteins by DARPin-E3 chimeras, H1299 cells were seeded into white Nunc 96-well microplates (Thermo Fisher Scientific) in triplicates. Twenty-four hours after seeding, the cells were transfected with plasmids containing the DARPin-E3 chimera were co-transfected with the pBit4.1-N[HiBit-IRES-luc2/CMV/Blast] vector containing the respective p63 or p73 isoform using Lipofectamine 2000 transfection reagent (Thermo Fisher Scientific) according to the manufacturer’s instructions. The firefly and Nanoluc luciferase activity was measured on a Spark plate reader (Tecan) using the Nano-Glo HiBit Dual-Luciferase Reporter System (Promega) according to the manufacturer’s recommendations. The experiment was repeated in three biological replicates. The protein level was determined by calculating the ratio of Nanoluc to firefly luciferase activity which was normalized to a sample not containing any DARPin-E3 chimera. Statistical significance was analyzed by ordinary one-way ANOVA (n.s.: *P* > 0.05, *P ≤ 0.05, **P ≤ 0.01, ***P ≤ 0.001, ****P ≤ 0.0001) using Prism (Version 8.2.1, GraphPad).

### Proteasome inhibition assay

Stable U-2 OS cells expressing TAp73α were seeded into 12-well plates. Expression was induced by supplementation of the culture medium with 1 µg/mL tetracycline (Thermo Fisher Scientific). Eight hours after induction of protein expression medium was exchanged to culture medium without antibiotics but supplemented with tetracycline, and cells were transfected with pcDNA3.1 (+) HA-NLS plasmids with the respective DARPin-E3 chimera N-terminally tagged with an HA-tag followed by a nuclear localization signal (NLS, sequence: KKKRKV). A sample transfected with empty pcDNA3.1 (+) served as negative control. Sixteen hours after transfection cells were treated with DMSO (negative control) or the proteasome inhibitor bortezomib (100 nM) for 6 h. Cells were detached with Accutase and resuspended in 1x LDS sample buffer followed by Western Blot analysis using the anti-myc (1:2000, clone 4A6, Millipore) antibody.

### Transactivation assay

For transactivation assays, H1299 cells were seeded in 12-well plates and transfected using Lipofectamine 2000 transfection reagent (Thermo Fisher Scientific) according to the manufacturer’s recommendations. All transfection mixes contained 200 ng of pRL-CMV plasmid (Promega) for constitutive expression of *Renilla* luciferase, 200 ng of a reporter plasmid with firefly luciferase under the control of the artificial pBDS-promotor (3 copies of the wt p53 binding site from the 14-3-3σ promotor) and varying amounts of pcDNA3.1 (+) HA-NLS with the respective DARPin-E3 chimera N-terminally tagged. The pBV-Luc BDS-2 3x WT plasmid (addgene plasmid #16515) was a gift from Bert Vogelstein [50]. In the case of the promotor squelching assay, the transfection mix contained 50 ng Myc-tagged ∆Np73α and 10 ng Myc-tagged p53 as well as the reporter plasmids and the DARPin-E3 chimera plasmid. For each assay, an empty vector control only comprising empty pcDNA3.1(+) was transfected to determine the fold induction without the presence of any DARPin. Twenty-four after transfection, cells were washed with PBS (Gibco), detached with Accutase and re-seeded into white Nunc 96-well microplates (Thermo Fisher Scientific) in quadruplicates. The assay was performed using the Dual-Glo Luciferase reporter assay kit (Promega) according to the manufacturer’s instructions, and firefly as well as *Renilla* luciferase fluorescence was measured using a Spark plate reader (Tecan). The remaining sample was centrifuged for 5 min at 500 *g*, pelleted cells were mixed with 1x SDS-loading buffer, and p53 protein levels were analyzed by western blot using the anti-myc (1:2000, clone 4A6, Millipore) antibody. The experiment was repeated in three biological replicates, and the ratio of firefly to *Renilla* luciferase signal was normalized to empty vector control for each biological replicate. Statistical significance was assessed by ordinary one-way ANOVA (n.s.: *P* > 0.05, *P ≤ 0.05, **P ≤ 0.01, ***P ≤ 0.001, ****P ≤ 0.0001) using Prism (Version 8.2.1, GraphPad).

### Statistics and reproducibility

Pulldown experiments, degradation assays and transactivation assays were performed in biological triplicates. All data points are shown in the corresponding figures with the bar diagrams presenting the mean value and the error bar presenting the SD. ITC measurements were performed twice, but the determination of the *K*_D_ value is based on a single measurement. The *K*_D_ values and the 95% confidence interval were determined by SEDPHAT [49]. Immunofluorescence stainings were performed in biological duplicates with exemplary images of one replicate shown in the respective figure.

### Ethical statement

All methods were performed in accordance with the relevant guidelines and Regulations. As this is an in vitro study without human patient material or experiments with animals no ethics committee approval was required.

## Supplementary information

original data

Supplementary Material

## Data Availability

The atomic coordinates and structure factors of the p73 OD complex with DARPin 1800 have been deposited in the Protein Data Bank (PDB) under accession code 9GLQ. The atomic coordinates and structure factors of the p73 SAM complex with DARPin B9 have been deposited in the Protein Data Bank (PDB) under accession code 9GNB.
