# Supplementary material for: DARPins as a novel tool to detect and degrade p73
Source: Cell Death Dis. 2024 Dec 18;15(12):909. doi: 10.1038/s41419-024-07304-2 (PMC11655841; doi:10.1038/s41419-024-07304-2)

Figure 1 - uncropped Western Blots - Boxes indicate area used in figure

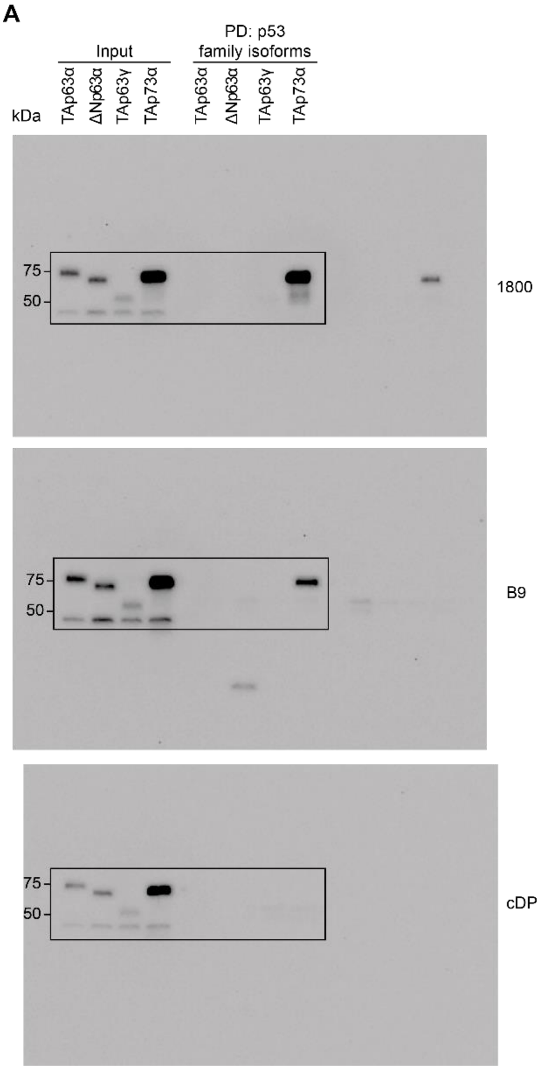


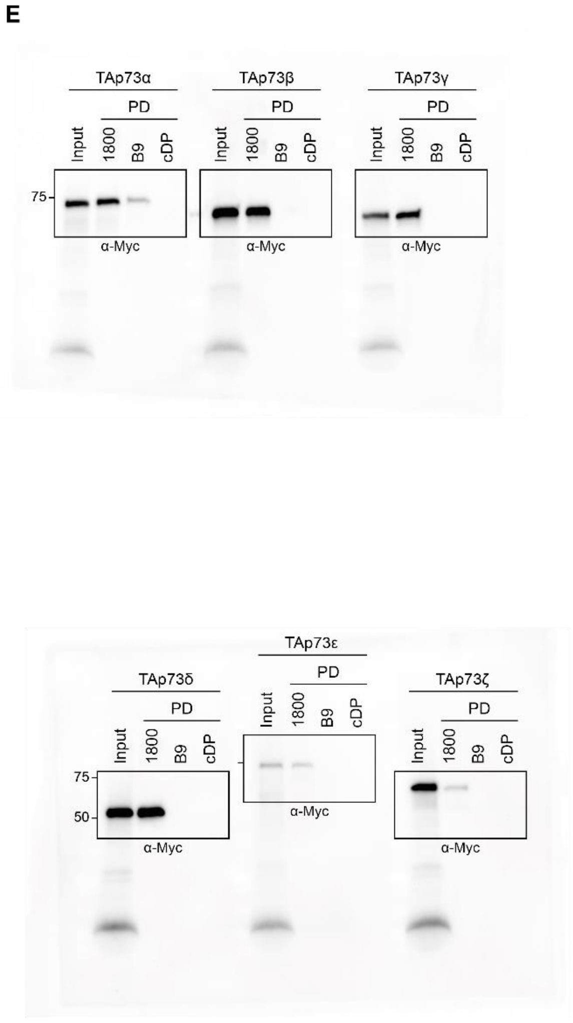

Figure 4 - uncropped Western Blots - Boxes indicate area used in figure

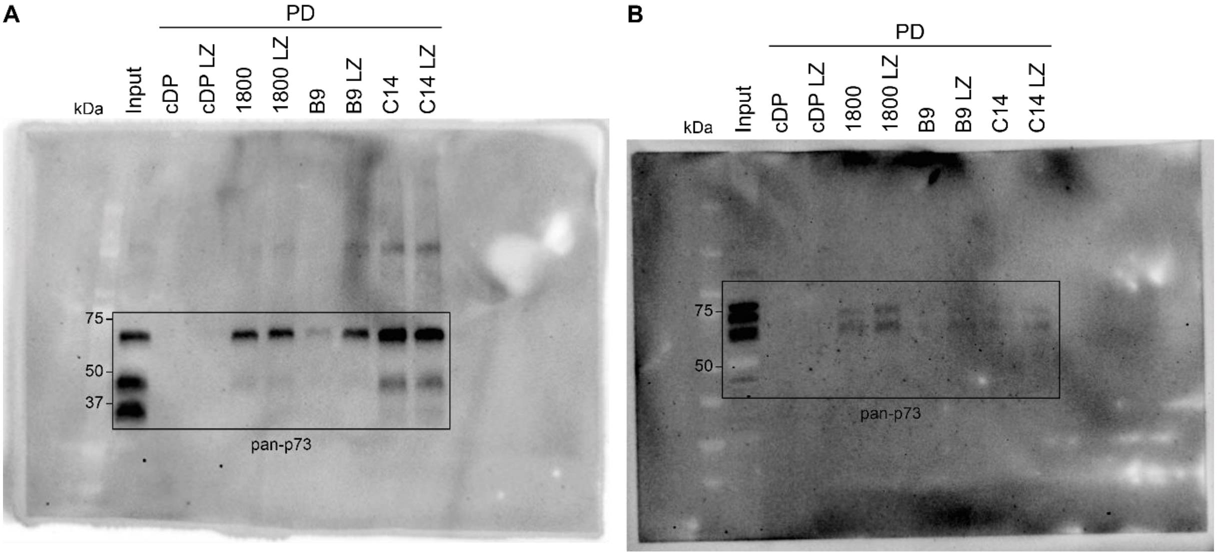

Figure 5 - uncropped Western Blots - Boxes indicate area used in figure

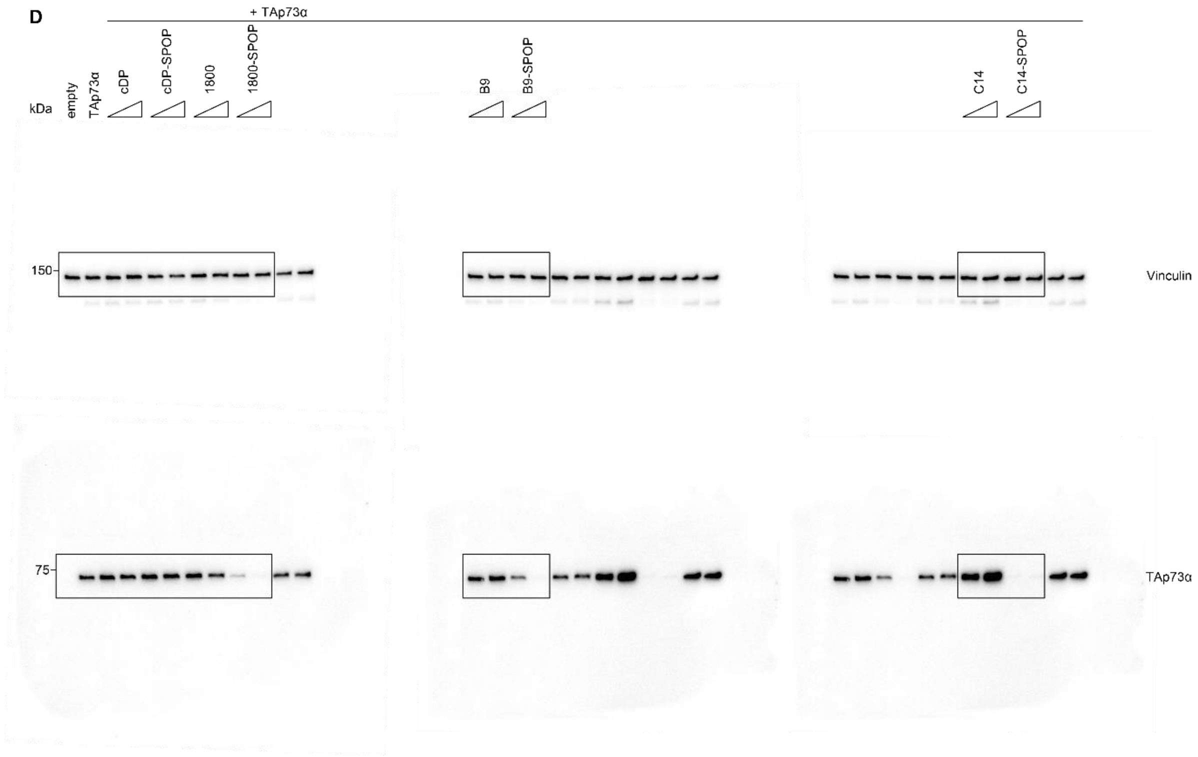

Figure 6 - uncropped Western Blots - Boxes indicate area used in figure

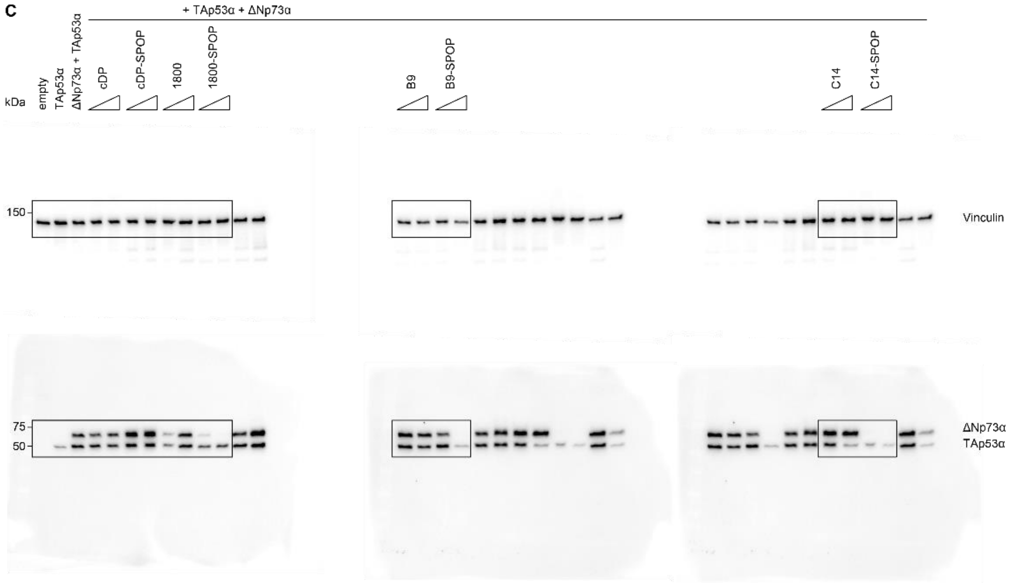


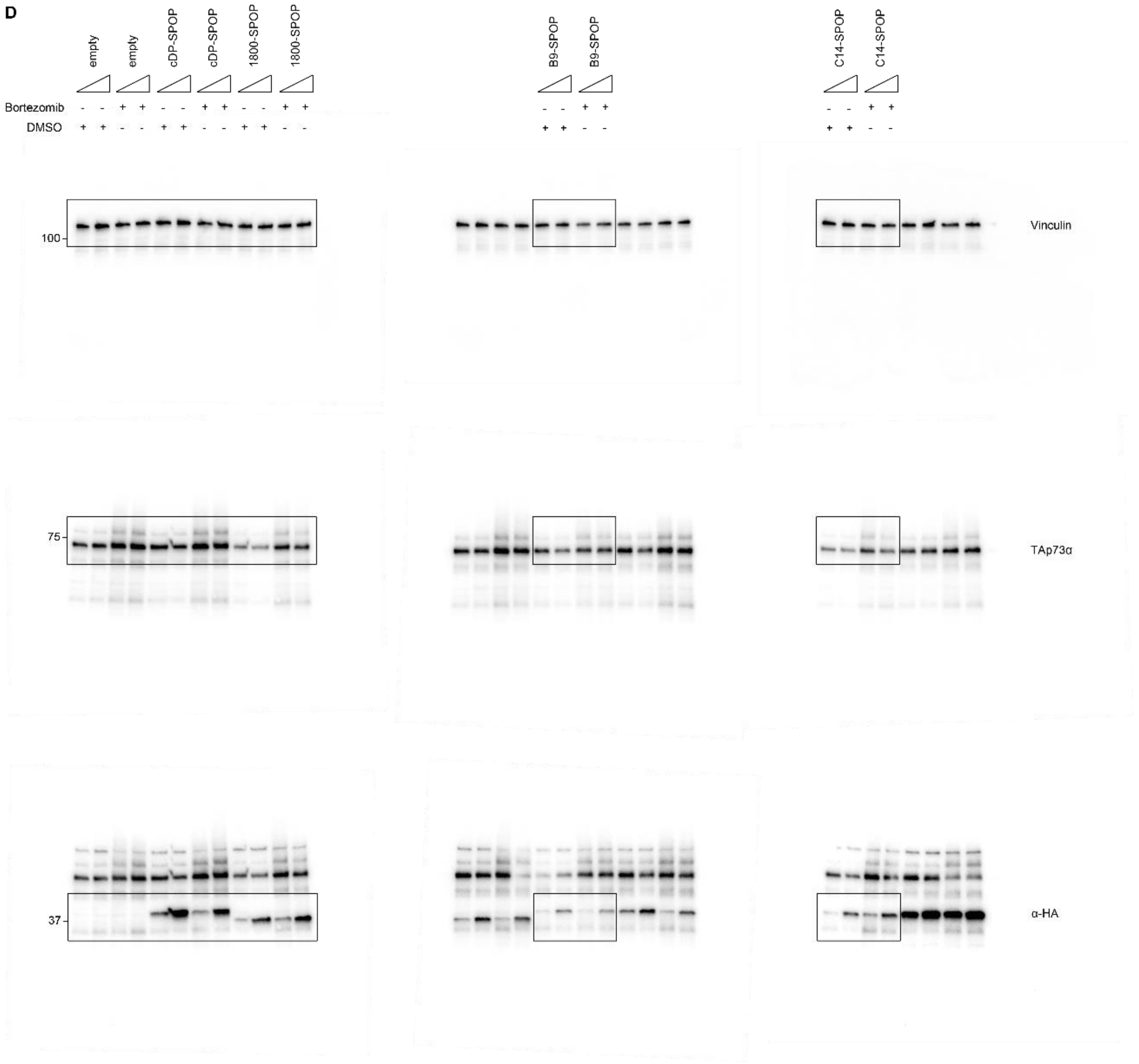

SupMat Figure 1 - uncropped Western Blots - Boxes indicate area used in figure

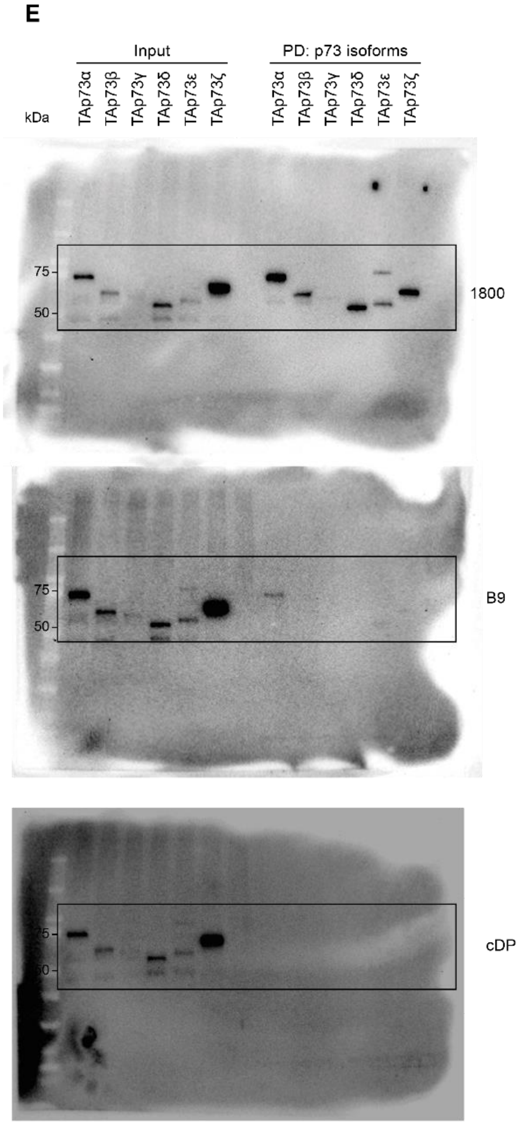

Supplement: Supplementary file 1 — original data [file 41419_2024_7304_MOESM1_ESM.pdf]
