# Supplementary material for: DARPins as a novel tool to detect and degrade p73
Source: Cell Death Dis. 2024 Dec 18;15(12):909. doi: 10.1038/s41419-024-07304-2 (PMC11655841; doi:10.1038/s41419-024-07304-2)
Supplement: Supplementary file 2 — Supplementary Material [file 41419_2024_7304_MOESM2_ESM.pdf]

SUPPORTING INFORMATION

DARPins: a novel tool to detect and degrade p73

Philipp Münick[1,*], Jasmin Zielinski[1,*], Alexander Strubel[1,*], Niklas Gutfreund[1], Birgit Dreier[3], Jonas V. Schaefer[3], Birgit Schäfer[1], Jakob Gebel[1], Christian Osterburg[1], Apirat Chaikuad[2,4], Stefan Knapp[2,4], Andreas Plückthun[3] and Volker Dötsch[1#]

[1]Institute of Biophysical Chemistry and Center for Biomolecular Magnetic Resonance, Goethe University, Frankfurt, Germany.

[2]Institute of Pharmaceutical Chemistry, Goethe University, 60438 Frankfurt, Germany.

[3]Department of Biochemistry, University of Zurich, 8057 Zurich, Switzerland

[4]Structural Genomics Consortium, Goethe University, Frankfurt, Germany.

*These authors contributed equally to this work
[#]Correspondence: vdoetsch@em.uni-frankfurt.de

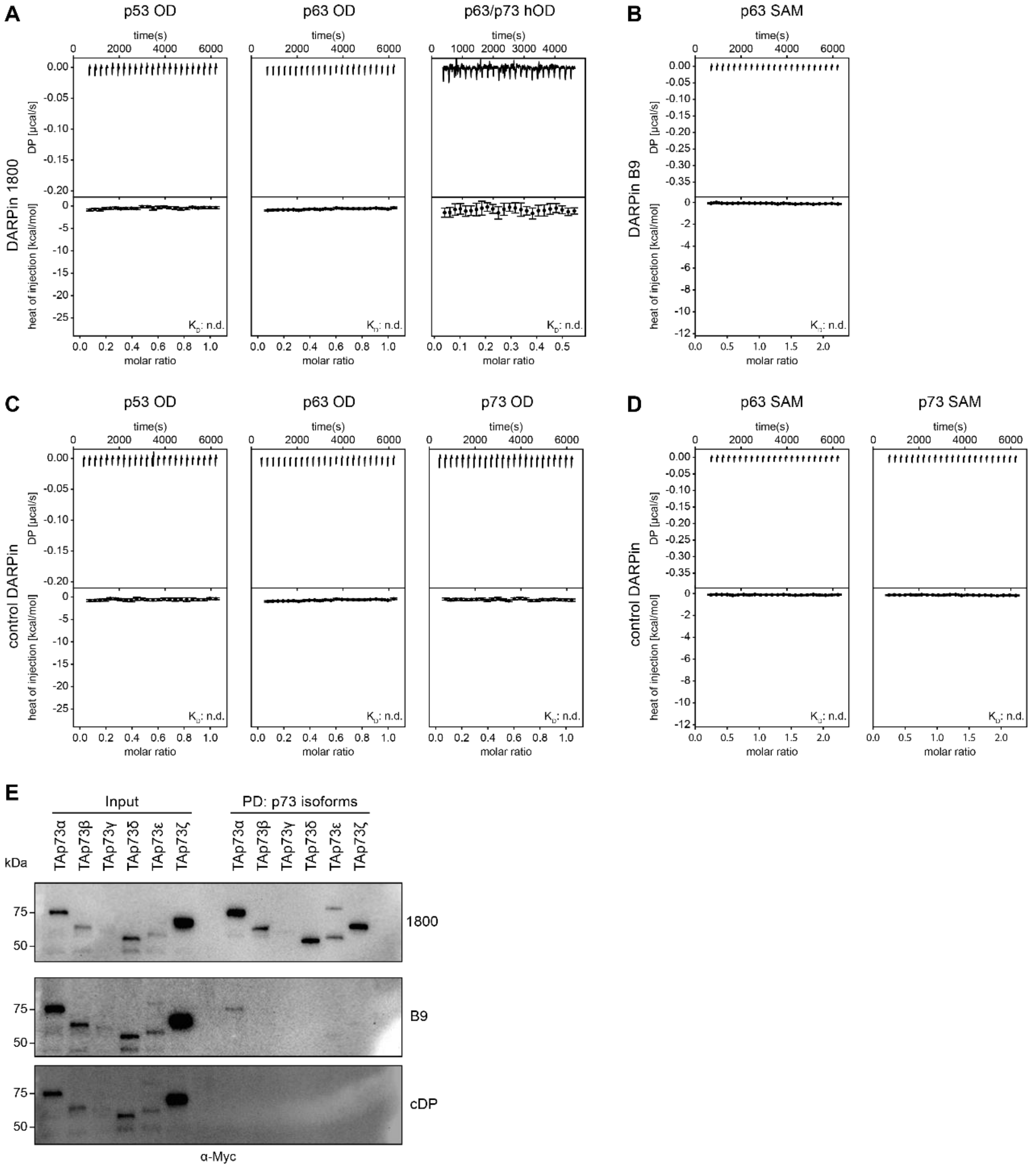

Supplementary Fig. 2 (A) ITC measurement with DARPin 6C9 and the ODs of the p53 family members p53 and p63 as well as with the p63$_2$/p73$_2$ hetero-TD performed at 25 °C. The top diagram shows the raw measurement and the bottom diagram the integrated heat per titration step. No interaction with either any of the ODs was observed, showing the high specificity of DARPin 6C9 for family member p73. **(B)** ITC measurement with DARPin B9 and the p63 SAM domain performed at 25 °C. The top diagram shows the raw measurement and the bottom diagram the integrated heat per titration step. No interaction can be observed showing the high specificity of DARPin B9 for p73. **(C)** ITC measurements with the control DARPin and the ODs of all p53 family members. **(D)** ITC measurements with the control DARPin and the SAM of all p53 family members. p53 does not contain a SAM domain and is therefore not included in the measurement. **(E)** Pulldown experiments with different Myc-tagged p73 isoforms transiently expressed in H1299 cells. Input signals are shown on the left, signals after pulldown on the right. The experiments were performed in biological triplicates with exemplary blots of one replicate shown.

A

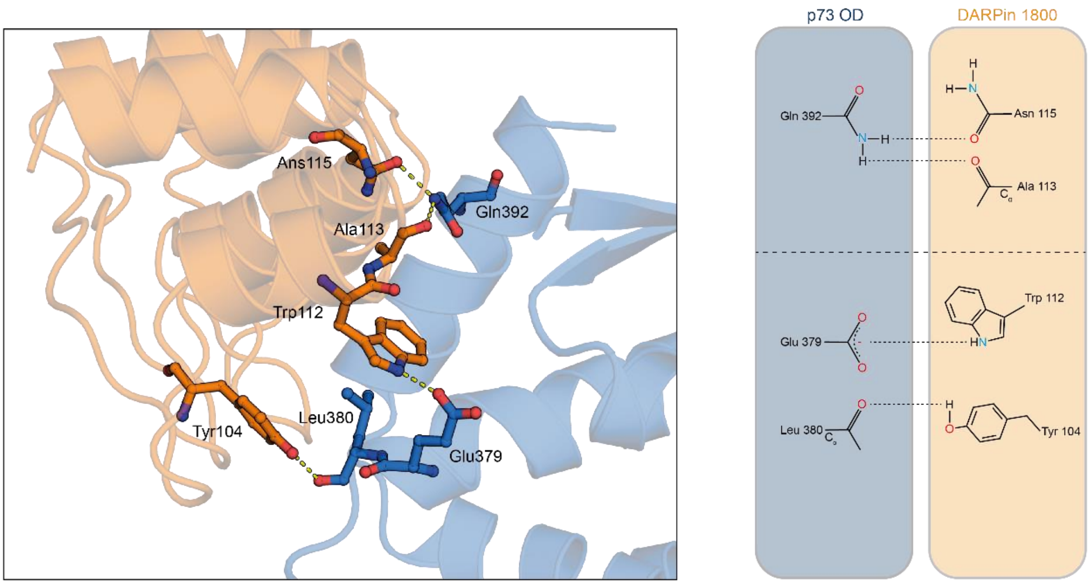

B

DARPin 1800
GSDLGKKLLEAAAVGQDDEVRILMANGADVNAMDQNGETPLHLAAMNGHLEIVEVLLKTGADVNASDFHGDTPLHLAAMAG
HLEIVEVLLKHGADVNAQDTWGYIPFDLAAWACNEDIAEVLQKAA

p73 OD
DEDTYYLQVRGRENFEILMKLKESLELMELVPQPLVDSYRQQQQLLQR
DEDTYYLQVRGRENFEILMKLKESLELMELVPQPLVDSYRQQQQLLQR

Hydrogen Bonds
Hydrophobic Contacts

C

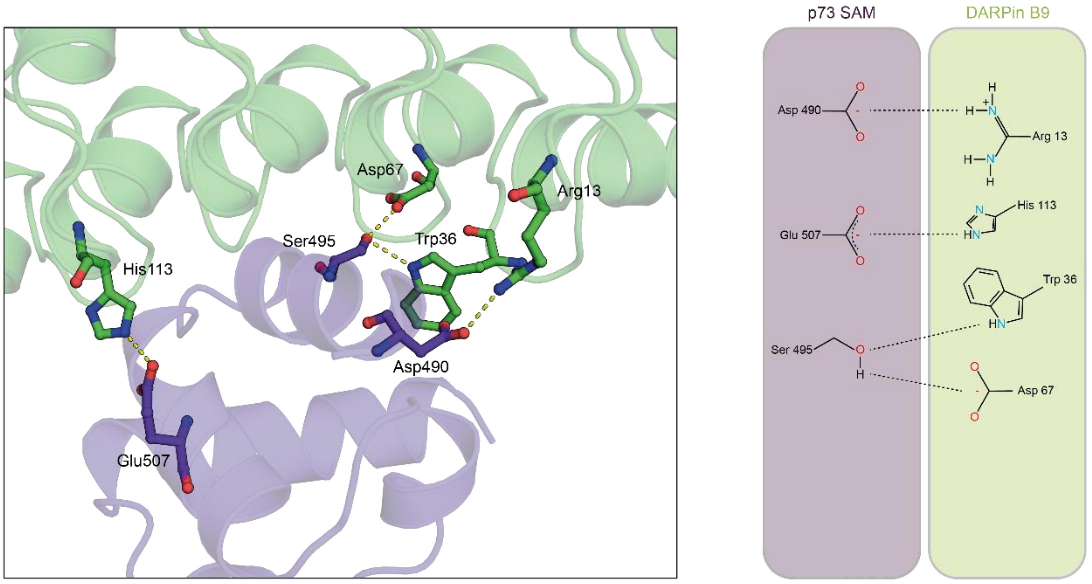

D

DARPin B9
GSDLGKKLLEAARAGQDDEVRILMANGADVNALDSWGFTPLHLAAFFGHLEIVEVLLKTGADVNAVDNAGTTPLHLAAHAG
HLEIVEVLLKAGADVNAHDQHGTVTPLHLAAHMGHLEIVEVLLKHGADVNAQDKFGKTPFDLAIDNGNEDIAEVLQKAA

p73 SAM
ADPSLVSFLTGLGCPNCIEYFTSQGLQSIYHLQNLTIEDLGALKIPEQYRMTIWRGLQDLKQ

Hydrogen Bonds
Hydrophobic Contacts

Supplementary Fig. 3 (A) Detailed analysis of the interface of DARPin 6C9 with the p73 OD analysed by LigPlot[ref]. The DARPin is colored orange and the p73 OD is colored blue. **(B)** Crucial amino acids of the interaction are also indicated within the sequences with hydrogen bonds highlighted in orange and hydrophobic contacts in green. **(C)** Detailed analysis of the interface of DARPin B9 with the p73 SAM analysed by LigPlot[ref]. The DARPin is colored green and the p73 SAM is colored purple. **(D)** Crucial

amino acids of the interaction are also indicated within the sequences with hydrogen bonds highlighted in orange and hydrophobic contacts in green.

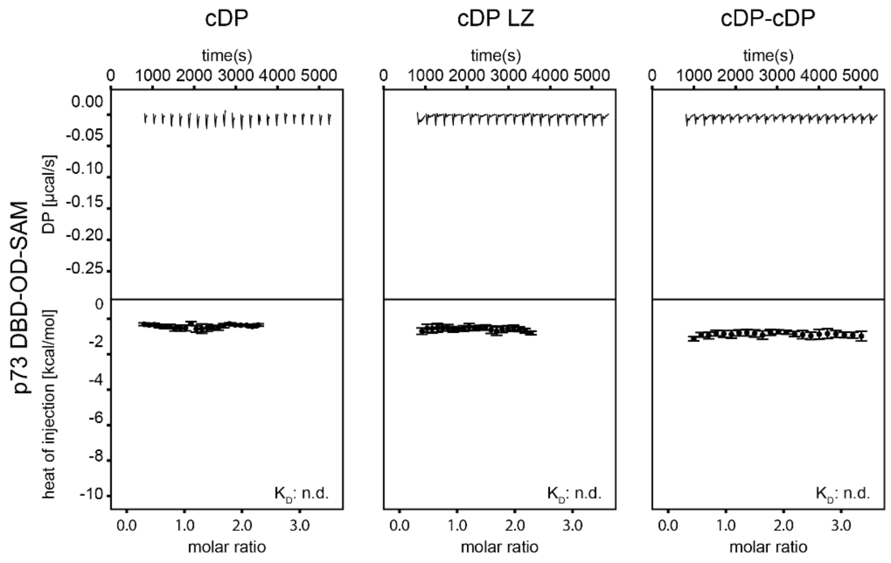

Supplementary Fig. 4 Interaction study of the p73 DBD-OD-SAM (amino acids 112-550 of TAp73α) and control DARPin (left panel), control DARPin dimerized via a leucine zipper (cDP LZ, middle panel) and a linear fusion of two control DARPins via a $(G_4S)_4$-linker (cDP-cDP, right panel) linker using ITC. The top diagram shows the raw measurement and the bottom diagram the integrated heat per titration step. The measurement was performed at 25 °C.

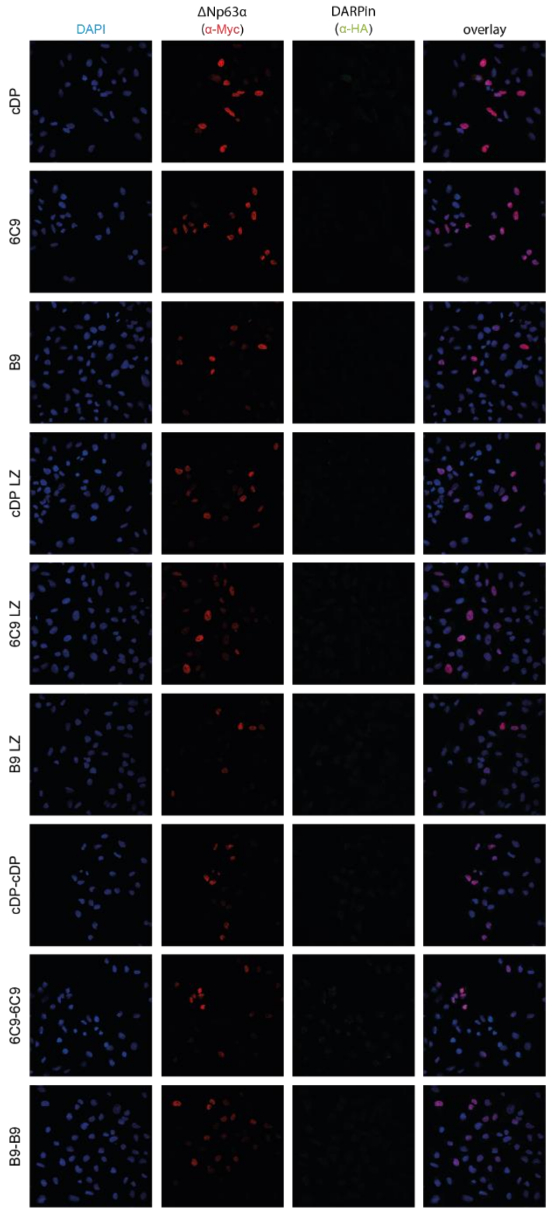

Supplementary Fig. 5 p73-binding DARPins do not detect p63 in stable expressing U-2-OS cells. Cells expressing**)** or ΔNp63α were fixed with formaldehyde and incubated with the indicated HA-tagged DARpin followed by incubation with goat anti-HA antibody (a190138a - Bethyl) and the secondary antibody Alexa Fluor 568 anti-goat (A11057—Life Technologies). The same cells were also incubated with mouse anti-myc antibody 4A6 (Millipore) and Alexa Fluor 647 anti-mouse antibody (A31571—Life Technologies) as both isoforms were Myc-tagged. None of the DARPin constructs shows any signal above background.

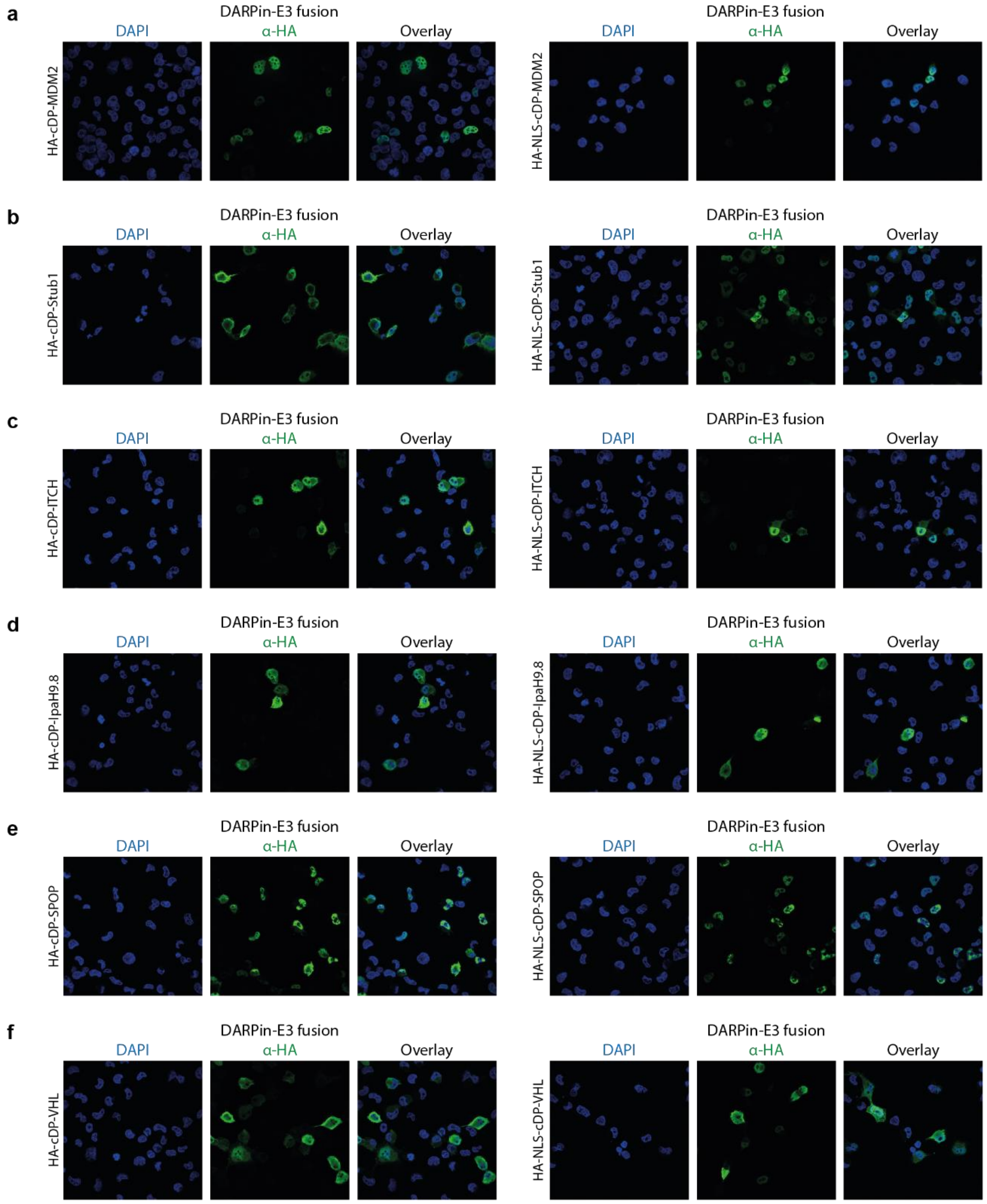

Supplementary Fig. 6 Analysis of the cellular localization of DARPin-E3 fusion constructs by immunofluorescence staining. The respective cDP-E3 fusion constructs either tagged with a N-terminal HA-tag or a N-terminal HA-tag followed by the SV40 NLS were transiently transfected in H1299 cells. 24 hours after transfection cells were fixed with formaldehyde and stained with goat anti-HA antibody (a190138a - Bethyl) and the secondary antibody Alexa Fluor 568 anti-goat (A11057—Life Technologies).

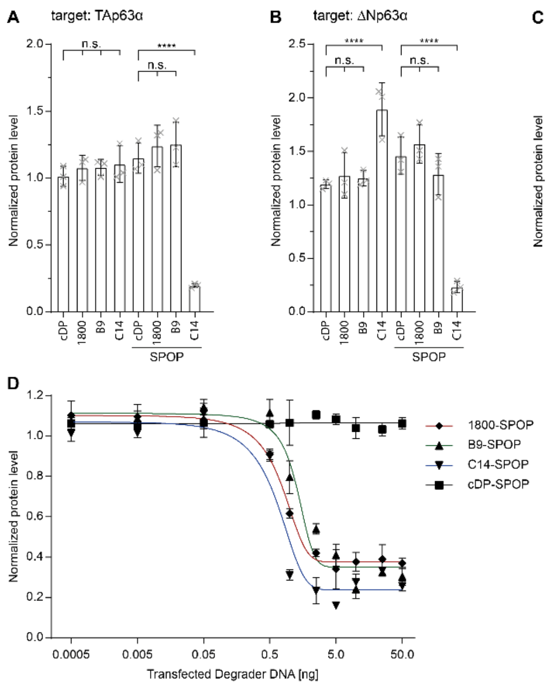

Supplementary Fig. 7 Investigation of the specificity of PROTACs based on DARPins binding to p73 domains. (A) Analysis of the protein level of HiBit-TAp63α when co-transfected with the DARPins 6C9, B9, C14 or cDP or the respective DARPin-SPOP fusion. Only DARPin C14 shows significant degradation of TAp63α**. (B, C)** Same experiment as in (A) but with HiBit-ΔNp63α (B) or HiBit-TAp53α (C). The bar diagrams in (A), (B) and (C) represent mean values of three biological replicates and the error bars the respective standard deviations presented.Statistical significance was assessed by ordinary one-way ANOVA (n.s.: P > 0.05, *: P ≤0.05, **: P ≤0.01, ***: P ≤0.001, ****: P ≤ 0.0001). **(D)** The degradation efficiency is dependent on the transfected amount of DARPin-SPOP fusion.

Supplementary Table S1. ITC statistics of p73 specific DARPins to their isolated target domains.

DARPin	Target	K_d [nM]	95% CI [nM]	ΔH [kcal mol^{-1}]	95% CI [kcal mol^{-1}]	ΔS [cal mol^{-1} K^{-1}]
1800	p53 OD	-	-	-	-	-
	p63 OD	-	-	-	-	-
	p73 OD	94.5	74.4 to 119.9	-30.3	-32.1 to -28.7	-69.4
	p63/p73 heteroOD	-	-	-	-	-
B9	p63 SAM	-	-	-	-	-
	p73 SAM	58.8	54.6 to 63.2	-11.4	-11.5 to -11.3	-5

Supplementary Table S2. Thermodynamic data of the ITC measurements performed with different DARPin constructs and the p73 DBD-OD-SAM construct. All measurements were performed at 25°C.

DARPin	K_d [nM]	95% CI [nM]	ΔH [kcal mol^{-1}]	95% CI [kcal mol^{-1}]	ΔS [cal mol^{-1} K^{-1}]
**1800**	67.2	38.7 to 109.4	-9.2	-9.2 to -8.8	1.9
**1800 LZ**	3.7	n.d. to 21.7	-6.5	-7.6 to -5.6	16.7
**1800-1800**	13.4	n.d. to 63.6	-8	-9.1 to -6.9	9.2
**B9**	309.0	199.0 to 490.5	-10.2	-11.4 to -9.3	-4.5
**B9 LZ**	64.1	47.5 to 84.7	-9.2	-9.5 to -8.9	0.6
**B9-B9**	45.9	28.5 to 70.4	-13.0	-13.8 to -12.4	-10.1

Supplementary Table S3. Data collection and structure refinement statistics

**Complex**	TP73A TD with DARPin 1800	TP73A SAM with DARPin B9
**PDB accession code**	**9GLQ**	**9GNB**
Data Collection		
Resolution[a] (Å)	48.22-2.10 (2.17-2.10)	67.98-1.80 (1.84-1.80)
Spacegroup	$I2_12_12_1$	$P12_11$
Cell dimensions	*a* = 62.8, *b* = 94.8, *c* = 96.4 Å<br>*α, β, γ* = 90.0	*A* = 48.3, *b* = 31.2, *c* = 68.4 Å<br>*α = 90.0, β = 95.97, γ* = 90.0
No. unique reflections[a]	17,152 (1,653)	18,890 (1,128)
Completeness[a] (%)	99.9 (100.0)	98.6 (99.4)
I/σI[a]	15.1 (2.0)	19.7 (3.6)
R_{merge}[a]	0.053 (0.913)	0.041 (0.342)
CC (1/2)	0.999 (0.938)	0.999 (0.942)
Redundancy[a]	7.5 (7.7)	6.7 (6.8)
Refinement		
R_{fact} (%)	20.2	20.1
R_{free} (%)	24.8	23.3
rms deviation bond[b] (Å)	0.008	0.01
rms deviation angle[b] (°)	1.2	1.6

[a] Values in brackets show the statistics for the highest resolution shells.
[b] rms indicates root-mean-square.
